# Vineyard microclimate alterations induced by black inter-row mulch through transcriptome reshaped the flavoromics of cabernet sauvignon grapes

**DOI:** 10.1186/s12870-024-04986-w

**Published:** 2024-04-09

**Authors:** Meng-Bo Tian, Yu Wang, Xiao-Tong Gao, Hao-Cheng Lu, Qi Zhang, Xiao Han, Hui-Qing Li, Ning Shi, Chang-Qing Duan, Jun Wang

**Affiliations:** 1 Center for Viticulture and Enology, College of Food Science and Nutritional Engineering, China Agricultural University, Beijing, 100083 China; 2 Key Laboratory of Jianghuai Agricultural Product Fine Processing and Resource Utilization, Ministry of Agriculture and Rural Affairs, Anhui Engineering Research Center for High Value Utilization of Characteristic Agricultural Products, College of Tea & Food Science and Technology, Anhui Agricultural University, Hefei, 230036 China; 3 Key Laboratory of Viticulture and Enology, Ministry of Agriculture and Rural Affairs, Beijing, 100083 China

**Keywords:** Vineyard management, Microclimate, Primary metabolites, Secondary metabolites, Transcription pathway

## Abstract

**Background:**

Weed control is essential for agricultural floor management in vineyards and the inter-row mulching is an eco-friendly practice to inhibit weed growth via filtering out photosynthetically active radiation. Besides weed suppression, inter-row mulching can influence grapevine growth and the accumulation of metabolites in grape berries. However, the complex interaction of multiple factors in the field challenges the understanding of molecular mechanisms on the regulated metabolites. In the current study, black geotextile inter-row mulch (M) was applied for two vintages (2016–2017) from anthesis to harvest. Metabolomics and transcriptomics analysis were conducted in two vintages, aiming to provide insights into metabolic and molecular responses of Cabernet Sauvignon grapes to M in a semi-arid climate.

**Results:**

Upregulation of genes related to photosynthesis and heat shock proteins confirmed that M weakened the total light exposure and grapes suffered heat stress, resulting in lower sugar-acid ratio at harvest. Key genes responsible for enhancements in phenylalanine, glutamine, ornithine, arginine, and C_6_ alcohol concentrations, and the downward trend in ε-viniferin, anthocyanins, flavonols, terpenes, and norisoprenoids in M grapes were identified. In addition, several modules significantly correlated with the metabolic biomarkers through weighted correlation network analysis, and the potential key transcription factors regulating the above metabolites including *VviGATA11*, *VviHSFA6B*, and *VviWRKY03* were also identified.

**Conclusion:**

This study provides a valuable overview of metabolic and transcriptomic responses of M grapes in semi-arid climates, which could facilitate understanding the complex regulatory network of metabolites in response to microclimate changes.

**Supplementary Information:**

The online version contains supplementary material available at 10.1186/s12870-024-04986-w.

## Background

Weed control is essential for agricultural floor management in vineyards, conventional permanent bare ground by continuous soil tillage can result in soil erosion, increasing soil disturbance and lowering organic matter. The use of herbicides increases the risk of damaging the ecological balance of the vineyard [1]. Alternatively, inter-row mulching is an eco-friendly practice to inhibit weed growth via filtering out photosynthetically active radiation (PAR), and besides weed suppression, inter-row mulch can influence grapevine growth and the accumulation of metabolites in grape berries [2–4]. In terms of light conditions, reflective mulches such as white or silver reflective films, and glass particles can increase the reflected PAR around the cluster zone, while the black or dark mulches can absorb the incident light and decrease the reflected solar radiation around the cluster zone [2, 3]. In parallel, the cluster-zone temperature could also be altered by inter-row mulching due to its influence on reflected solar radiation.

Previous studies reported the effects of inter-row mulching on grape composition, especially in cool climate regions. In general, reflective mulch shows a tendency to promote grape ripening, and the accumulation of sugars and phenols due to the increased reflective solar radiation. Some studies reported that although reflective inter-row mulching increased reflective solar radiation, no significant influence on grape composition including total soluble solids, titratable acidity, pH, total phenols, anthocyanins, and flavonols was found [2, 3, 5]. The inconsistent effects of mulching treatments on grape composition could be attributed to the complex interactions between mulching materials, grape cultivars, soil properties, trellis system, vineyard management, and vintage climates. Therefore, the mulching strategy needs to be adjusted based on investigations on the plasticity of specific grapes to local terroir and vintage climate characteristics. So far, little information is available regarding the grape performance in dry and hot regions in response to inter-row mulching. Based on the gap above, our previous studies investigated the comprehensive effects of black geotextile inter-row mulching on Cabernet Sauvignon vine growth and grape composition in a semi-arid wine region. In addition, we undermined the potential causes of the influence of black inter-row mulching on grape composition through correlation analysis between metabolite concentrations and climatic parameters [4, 6].

The grapevine microclimate variations on metabolite accumulation in grapes could be attributed to multiple factors such as light, temperature, water status, soil nutrients status, etc. The regulation mechanism of light on metabolite accumulation has been extensively reported. Many studies revealed that sunlight exposure upregulated the expressions of key genes involved in the biosynthesis of anthocyanins, flavonols, terpenoids, and norisoprenoids, such as *CHS*, *DRF*, *F3H*, *LDOX*, *UFGT*, *AOMT*, *FLS*, *CCD*, *TPS*, etc [7–10]. . . As for temperature effects, moderate temperature is favorable for anthocyanin accumulation, while high temperature could decrease anthocyanins [11, 12]. Regarding volatile compounds, it was reported that relatively low temperatures and high temperatures both upregulated the expressions of *VviCCDs*, which led to increases in norisoprenoids in grapes [13]. As for water status, many studies investigated the effects of deficit irrigation level and timing on grape composition. Generally, moderate deficit irrigation at the green stage was considered to be favorable for berry ripening and the accumulation of flavor compounds which was not only ascribed to the decreases in berry volume but also the upregulation of relevant key structural genes [14, 15].

Extensive variation in vineyard environments leads to the phenotypic plasticity of grapevines within and across vintages. Under field conditions, the influence of inter-row mulching on grape composition was ascribed to the complex interaction of multiple factors including light, temperature, soil temperature, and soil water content, and the vintage interventions. Particularly, the two key factors, light, and temperature, are often difficult to separate under field conditions. However, inter-row mulch diminishes light in the cluster zone and increases temperature, and becomes an optimal treatment to separate these two factors [4]. Besides, the interaction effect of multiple environmental factors on the global metabolites of grapes and the underlying molecular mechanism is still limited. In this study, we conducted metabolomics and transcriptomics analyses in two vintages, aimed at providing insights into metabolic and molecular responses of Cabernet Sauvignon grapes to microclimate changes caused by black inter-row mulching in a semi-arid climate, and assisting winegrowers to adjust agricultural strategies to cope with the interactions of multi-stress field environment.

## Results

### The transcriptomic changes of grape berries under inter-row mulching

The overview of transcriptomic profiles of grape berries was presented in Text S1. The linear regression analysis between the expressions assessed by qRT-PCR and RNA sequencing validated that expression profiles obtained by RNA sequencing were reliable (Fig. S1).

In total, 717/485, 1736/1869 and 11/13 genes were up/down-regulated by inter-row mulching treatment at E-L 33, E-L 35.5, and E-L 38 stages respectively in 2016, and 190/144, 44/25 and 7/2 genes were up/down-regulated by inter-row mulch at E-L 33, E-L 35.5, and E-L 38 stages respectively in 2017 (Fig. S2). The numbers of DEGs between M and C at E-L 33 and E-L 35.5 stages were higher than those at E-L 38. Besides, the number of differentially expressed genes (DEGs) at E-L 33 and E-L 35.5 stages in 2016 was far higher than those in 2017.

### Consistent DEGs revealed the variables of microclimate and physiology under inter-row mulching

**Fig. 1:**
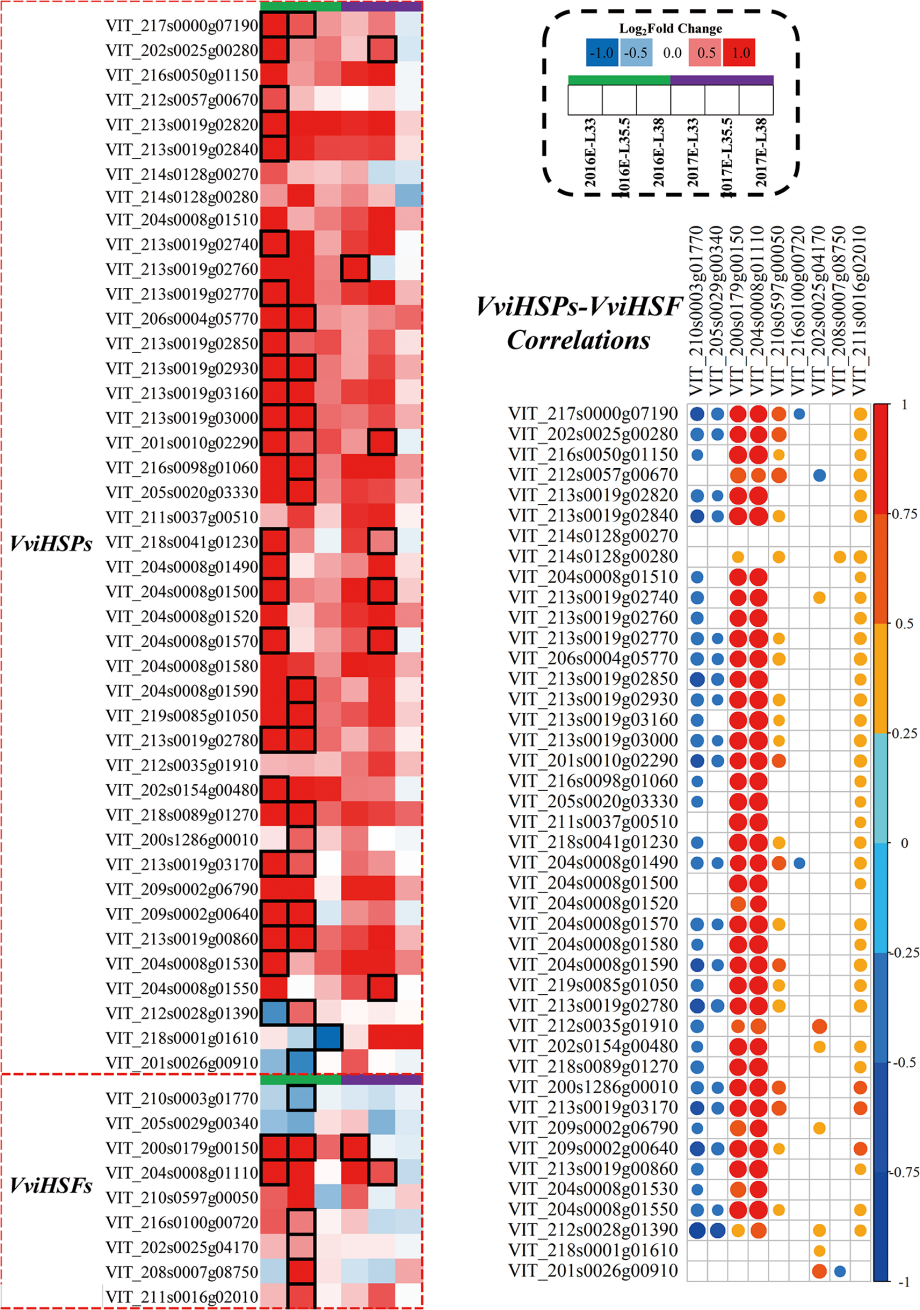
Effects of inter-row mulching on expression profiles of genes related to heat shock protein, and correlation analysis between heat shock transcription factors and heat shock protein. Data of gene expression heatmap represents the log_2_-transformed fold changes of gene expressions between inter-row mulching and control group. The black border of the heatmap cell represents significant differences in gene expressions between the inter-row mulching and control group (student’s t-test, *p* < 0.05). The color of each cell (blue to red) in the correlation heatmap represents the correlation coefficient (-1 to 1). The same as below

At E-L 33 stage, the Venn plot showed that inter-row mulching significantly upregulated the expression of heat shock transcription factor HSFA6B at E-L 33 stage in two years (Fig. S3). Specifically, M upregulated *VviHSF18* (VIT_204s0008g01110), *VviHSF02* (VIT_200s0179g00150), and *VviHSF01* (also known as *VviHSFA6B*, VIT_210s0597g00050) in two vintages (Fig. 1). Consistently, 37 heat shock proteins in total presented higher level in M than C grapes in both vintages. Further correlation analysis on *VviHSP* and *VviHSF* showed that *VviHSF02* and *VviHSF18* had the strongest correlation with *VviHSP*. In terms of 12 downregulated genes, transcription factor TSRF1 involved in the ethylene-mediated signaling pathway, as well as the photosystem II protein D2 involved in photosynthesis were negatively affected by M in both vintages (Fig. S3). At veraison, M significantly upregulated 23 genes in two years (Fig. S4), including genes related to photosynthesis, and genes encoding NADPH: protochlorophyllide oxidoreductase in chlorophyll biosynthesis.

The further *k*-means and KEGG analysis were conducted based on DEGs identified in each vintage respectively, which were described in Text S2. Overall, based on the DEGs in two years and the microclimate we previously reported [4], we concluded that genes related to photosynthesis and heat shock proteins in grapes at E-L 33 and 35.5 stages were upregulated by M, while C grapes experienced higher light exposure and severe drought stress. Besides, the influences of gene expression in grapes were highly dependent on vintages, and thus limited consistent DEGs were identified in two vintages.

### The profiles of sugars and organic acids revealed the ripening difference under inter-row mulching

To investigate the effects of inter-row mulching on grape composition, untargeted metabolome analysis was conducted. In this study, M slightly inhibited the accumulation of glucose, fructose, and sucrose at E-L 33, but accelerated sugar accumulation at veraison (Table 1). At harvest, most sugars presented at a lower level than C grapes, especially in 2017. M also inhibited most of the organic acids at E-L 33, especially the citric acid and malic acid in 2017, however at harvest, M grapes presented higher levels of organic acids in both vintages.

**Table 1:** Log2 fold change of relative contents of sugars and organic acids

Metabolites	2016			2017		
	E-L 33	E-L 35.5	E-L 38	E-L 33	E-L 35.5	E-L 38
D-Fructose	-0.17	0.82	0.22	-1.35	0.41	-0.27
D-Glucose	-0.98	0.67	0.16	-0.36	0.14	-0.37*
Sucrose	-0.59	-0.50	-1.02	-0.80	0.44	-1.35
Tartaric acid	-0.13	-0.01	0.35	-0.13	-0.07	-0.07*
Malic acid	-0.08	-0.39	0.58	-0.37*	-0.27	0.32
Citric acid	0.29	-0.37	0.10	-0.32*	-0.38	-0.08
*cis*-Aconitate	0.15	-0.44	0.11	-0.34*	-0.41	-0.11
Fumaric acid	-1.52*	-1.15	-2.92	-0.30	-0.19	-1.89
Succinic acid	-0.46*	-0.85*	0.14	-0.06	-0.60*	-0.40*

Note: The relative contents (Control vs. inter-row mulching) were calculated by the ion’s intensity, ‘*’ represents a significant difference at *p* < 0.05 (student’s *t*-test)

### Grapes of inter-row mulching accumulated more amino acids

Regarding the amino acids involved in glutamate metabolism (Fig. S5), M showed a tendency to increase the concentrations of glutamate, glutamine, ornithine, arginine, and proline at veraison and harvest. In particular, the concentrations of ornithine and arginine in M grapes were significantly higher than in C grapes at veraison in 2016, and so were the glutamate at veraison in 2017, and proline at veraison in both vintages. In amino acids of the shikimate pathway, M tended to increase the concentrations of tryptophan and tyrosine at E-L 33 stage. At veraison, M slightly decreased the tryptophan concentrations, whereas significantly increased phenylalanine concentrations. At harvest, M showed a tendency to increase the concentrations of tryptophan and phenylalanine. As for aspartate metabolism, M had no significant influence on the concentrations of asparagine, aspartate, and lysine at veraison and harvest in two vintages.

**Fig. 2:**
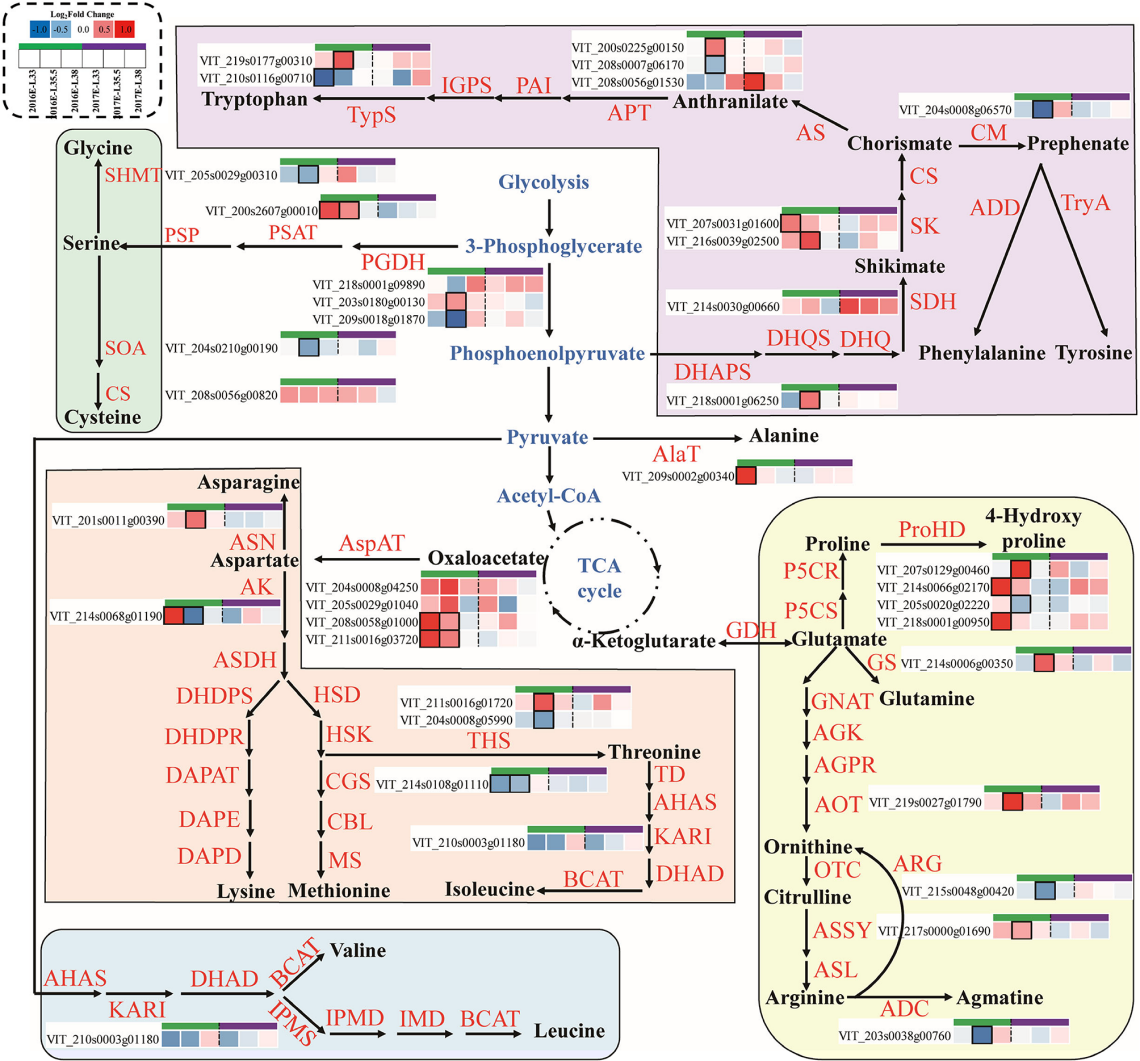
Effects of inter-row mulching on expression profiles of genes related to amino acid metabolism

At the transcription level, M significantly upregulated the expressions of *VviGS*, *VviASSY*, and *VviAOT* at veraison in 2016 and showed a similar upregulation tendency in 2017 (Fig. 2). In terms of aromatic amino acids, the 3-deoxy-D-arabinheptanoic acid-7-phosphate (DHAP) synthase (DHAPS) gene *VviDHAPS*, *VviSDH*, and 2 shikimate kinase genes were upregulated by M at veraison in 2016, most of them also presented at higher level than C grapes in 2017 (Fig. 2). Besides, M significantly downregulated *VviCM* at veraison in 2016 and showed a similar tendency in 2017.

### The inter-row mulching downregulated genes related to the synthesis, modification, and transport of phenylpropanoid metabolism

**Fig. 3:**
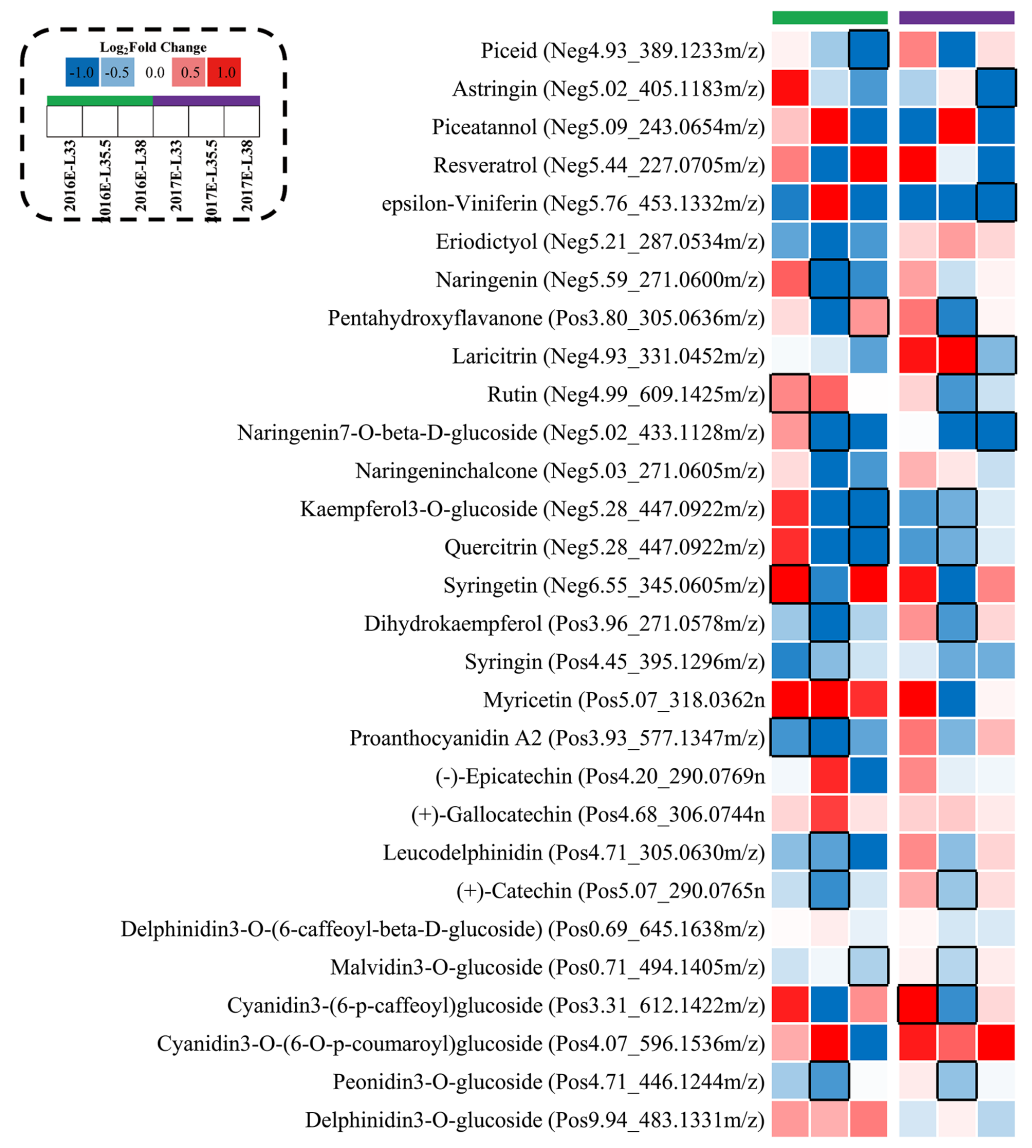
Effects of inter-row mulching on metabolites in phenylpropanoid pathway

In metabolites of the phenylpropanoid pathway, five stilbenes were identified in grapes, including piceid, astringin, piceatannol, resveratrol, and *ε*-viniferin (Fig. 3). Most stilbenes were enhanced by M at E-L 33 stage, whereas they presented at a lower level at harvest. Notably, M showed a tendency to decrease *ε*-viniferin concentrations at E-L 33 and 38 stages in 2016 and at three stages in 2017. These results suggested that M inhibited the accumulation of stilbenes in grapes post veraison. Regarding flavonoids, M decreased the concentrations of kaempferol-3-*O*-glucoside, quercitrin, and syringing at veraison and harvest, while having little influence on flavan-3-ols. In terms of anthocyanins, M significantly decreased malvidin-3-*O*-glucoside concentrations at harvest in 2016, and it also showed a tendency to decrease the concentrations of acetylated anthocyanins. However, M had little influence on anthocyanins in grapes in 2017.

**Fig. 4:**
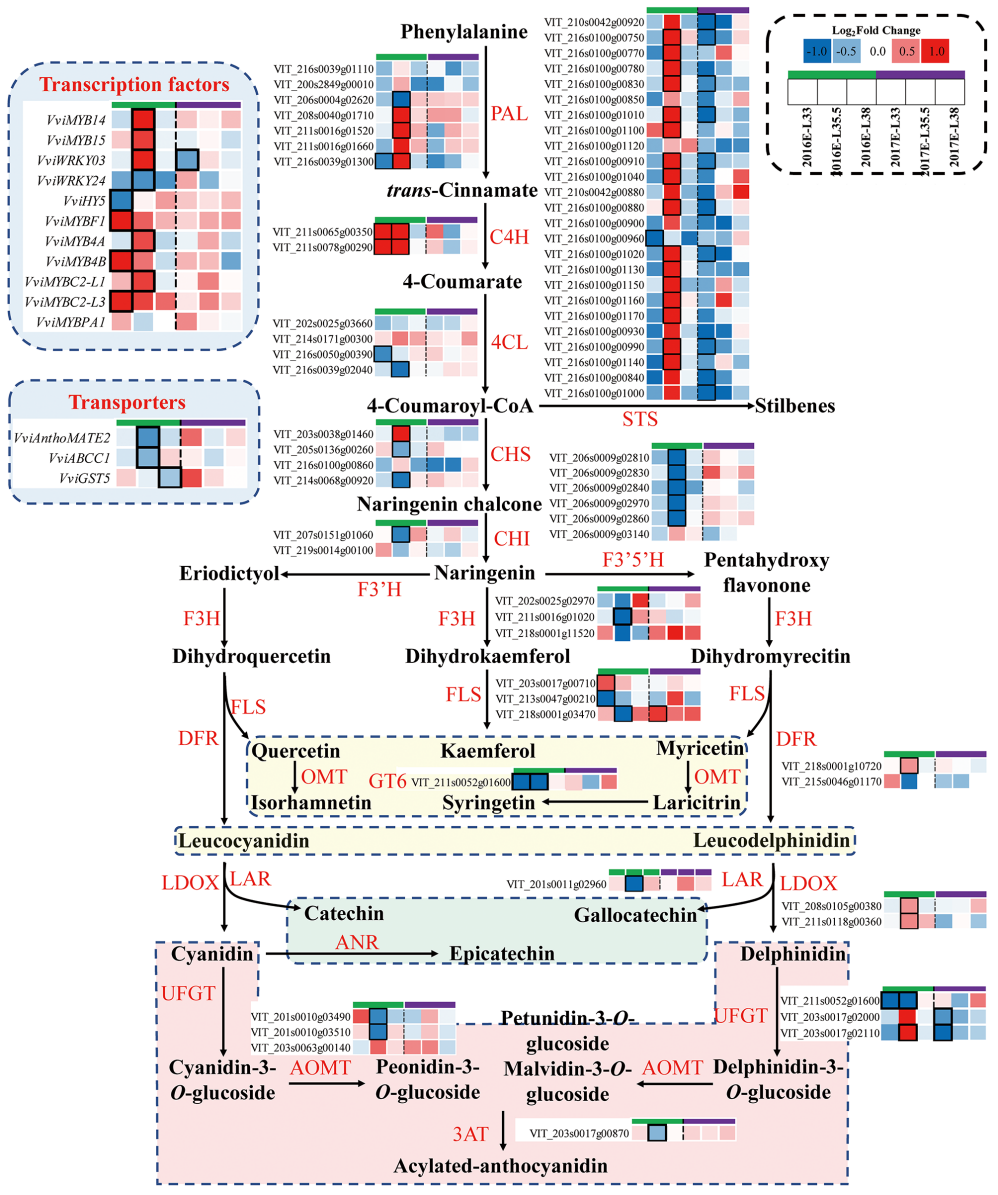
Effects of inter-row mulching on expression profiles of genes in phenylpropanoid metabolism

At the transcriptomic level. M showed downregulation tendency for 2 *VviPAL* genes (VIT_216s0039g01110, VIT_216s0039g01300) at E-L 33 stage, whereas upregulated 3 other *VviPAL* genes (VIT_208s0040g01710, VIT_211s0016g01520, VIT_211s0016g01660) at veraison (Fig. 4). M upregulated *VviC4H* expressions in 2016, and they also showed a similar tendency at E-L 33 stage in 2017. However, *Vvi4CL*, *VviCHS*, and *VviCHI* presented at a lower level in E-L 33, and/or E-L 35.5 in two years. Stilbene synthase (STS) is a key enzyme for the biosynthesis of stilbenes. Notably, STSs were negatively affected by M in most of the phenology stages, however, it presented a significant higher level than C grapes at veraison in 2016.

In the flavonoid pathway, M downregulated 5 *VviF3’5’H* genes in 2016, with the most significant influence observed at veraison (Fig. 4). In flavonol synthesis, M significantly downregulated 3 *VviF3H* genes, *VviFLS4* (VIT_218s0001g03470), and *VviGT6* at E-L 35.5 in 2016. In anthocyanin metabolism, M significantly upregulated the expression of *VviLDOX* in 2016, two genes encoding UFGT at E-L 35.5 in 2016, but significantly downregulated their expression in 2017. Anthocyanin acyltransferase (3AT) controls the biosynthesis of stable acylated anthocyanins. In this study, M significantly downregulated *Vvi3AT* at veraison in 2016.

Regarding the transportation of the anthocyanins, M downregulated the genes encoding *AnthoMATE2*, *VviABCC1*, and *VviGST 5* at veraison or at harvest (Fig. 4). The expressions of *VviGST4* and *VviGST5* in grapes were relatively low at E-L 33 stage while drastically increased at veraison (FPKM range in 300–600), and slightly declined at harvest. However, M had no significant influence on *VviGST4* expressions, but the expressions of *VviGST5* in M grapes were significantly lower than in C grapes at harvest in 2016.

For the transcription factors reported, M significantly upregulated the expressions of MYB family, including *VviMYB14*, *VviMYB15*, *VviMYBF1, VviMYB4A*, *VviMYB4B*, *VviMYBC2-L1*, *VviMYBC2-L3*, and *VviWRKY03* at E-L 33 and/or veraison in two vintages (Fig. 4). In addition, M tended to downregulate *VviWRKY24* and *VviHY5* at veraison and E-L 33 stage in 2016, respectively.

### The inter-row mulching inhibited MEP synthesis of isoprenoid metabolites and limited glycosylation process

**Fig. 5:**
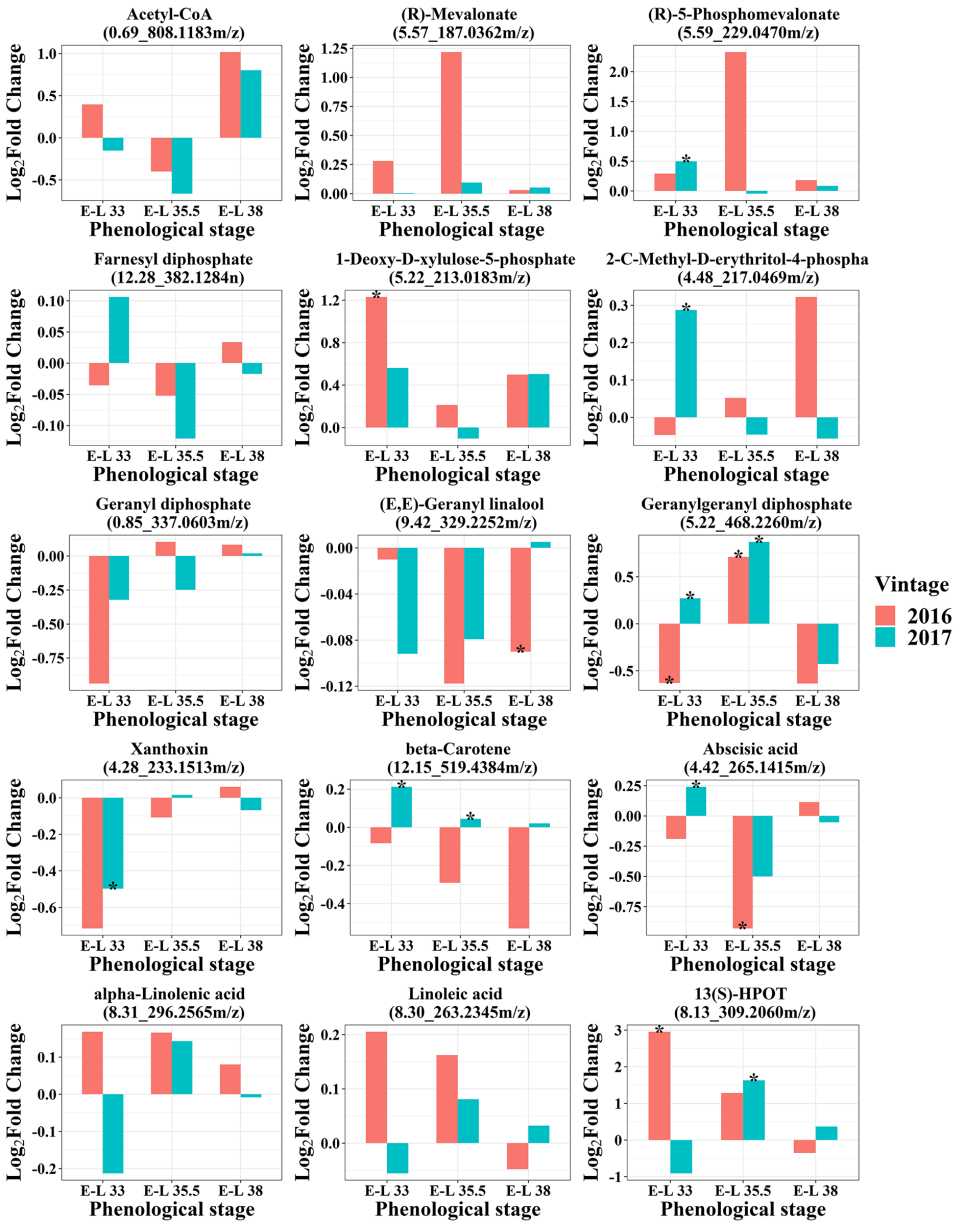
Effects of inter-row mulching on metabolites in terpenoids biosynthesis, carotenoid metabolism, and lipoxygenase pathway

In the terpene biosynthesis pathway, M showed a tendency to decrease (*E*,*E*)-geranyl linalool concentrations which was significantly lower in M grapes than in C grapes at harvest in 2016 (Fig. 5). Carotenoid metabolism relates to the terpene biosynthesis pathway via geranylgeranyl diphosphate (GGPP), which is a precursor of norisoprenoids. In this study, M significantly increased GGPP concentrations at veraison, while decreasing its concentrations at harvest. In terms of other metabolites in carotenoid metabolism, M decreased *β*-carotene levels in 2016, while the opposite results were observed in 2017. As for xanthoxin, M tended to decrease its level at E-L 33 stage, especially since its level in M grapes was significantly lower than in C grapes in 2017, while M had little influence on xanthoxin at veraison and harvest.

**Fig. 6:**
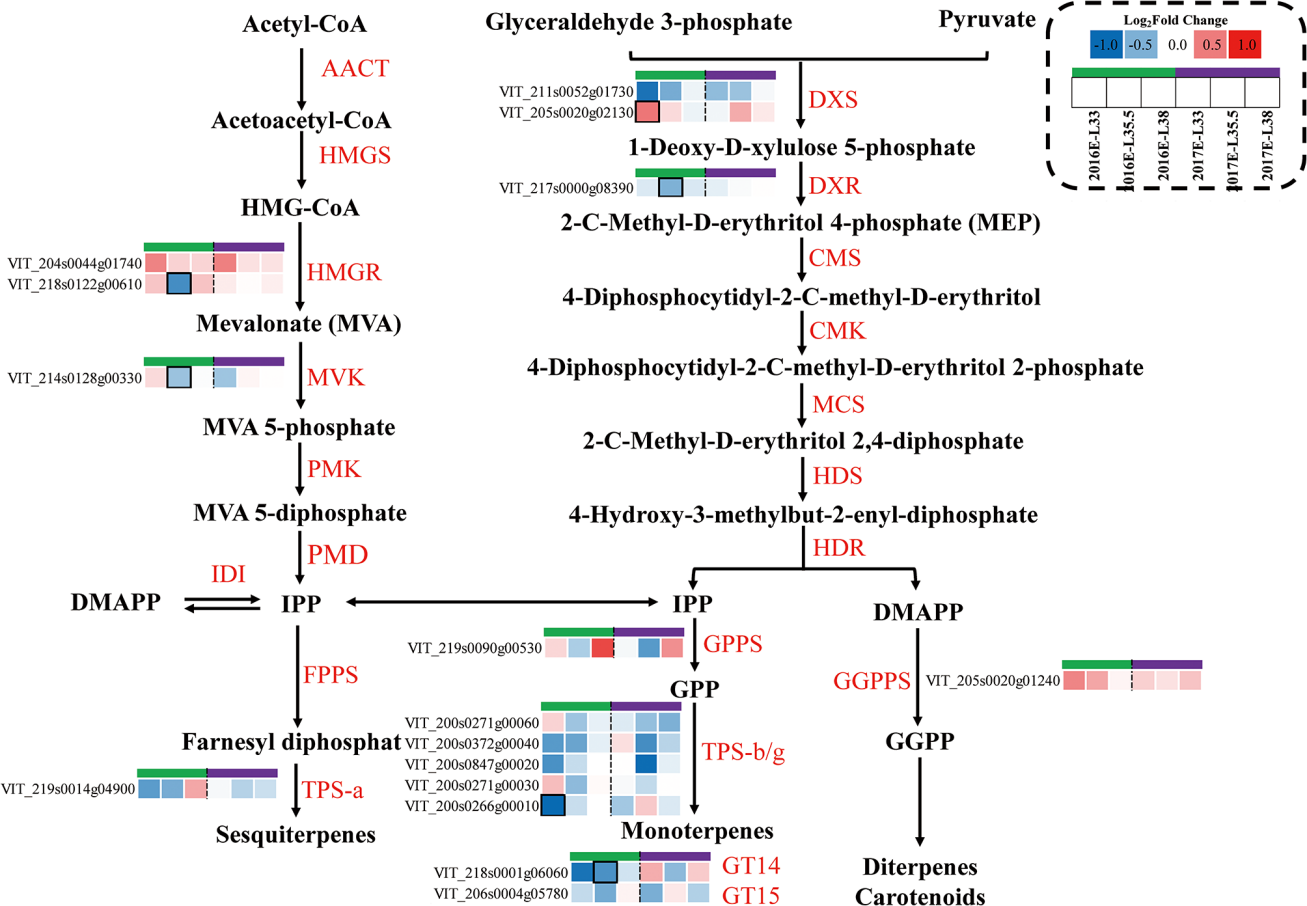
Effects of inter-row mulching on expression profiles of genes in terpenoid biosynthesis pathway. Expression profiles of genes related to carotenoid metabolism are shown in Fig. S6

At the transcriptomic level, M upregulated a gene encoding DXS (VIT_205s0020g02130) at 35.5 stages in both vintages (Fig. 6), whereas *VviDXR* and *VviGPPS* were significantly downregulated by M at veraison in 2016. In terms of terpene synthases. At E-L 33 stage, M downregulated three *VviTPS*-*b/g* genes in 2016, and 4 were also downregulated in both vintages at E-L 35.5. M also downregulated a *VviTPS*-*a* gene at E-L 33 and 35.5 stages in 2016, and at E-L 35.5 and 38 stages in 2017. In grapes, a large fraction of terpenoids are present as nonvolatile terpene glycosides, M showed a tendency to downregulate *VviGT14* and *VviGT15* at E-L 33 and 35.5 stages in 2016, particularly since the expressions of *VviGT14* in M grapes at veraison in 2016 were significantly lower than those in C grapes. In 2017, M moderately downregulated *VviGT14* at veraison, and *VviGT15* at E-L 33 and 38 stages.

In carotenoid metabolism, M showed a tendency to upregulate *VviGGPPS* at three developmental stages in 2016 and 2017 (Fig. 6), as well as *VviLECY* (Fig. S6). ABA biosynthesis pathway is a downstream flux of carotenoid metabolism, M downregulated 2 genes (VIT_210s0003g03750, VIT_219s0093g00550) encoding 9-*cis*-epoxycarotenoiddioxygenase (NCED) at E-L 33 and E-L 35.5 in 2016. However, in 2017, M showed a tendency to upregulate three NCED genes (VIT_210s0003g03750, VIT_219s0093g00550, VIT_205s0051g00670).

The biosynthesis of norisoprenoids is another downstream flux of carotenoid metabolism, in which carotenoid cleavage dioxygenases (CCD) catalyze the cleavage of carotenoids at certain positions to release norisoprenoids. M significantly upregulated *VviCCD1* (VIT_213s0064g00840) and moderately upregulated *VviCCD4a* (VIT_202s0087g00910) at veraison in 2016 (Fig. S6).

ABA as a plant hormone, is a key endogenous messenger in responses to abiotic stress. In this study, M significantly upregulated 2 ABA receptor *VviPYR/PYL* genes (Fig. S7), while it significantly downregulated 17 type 2 C protein (PP2C) *VviPP2C* genes at veraison in 2016. Besides, the expressions of 13 out of 17 *VviPP2C* genes were lower in M grapes than in C grapes in 2017.

### The inter-row mulching enhanced the C6 alcohols by regulating alcohol dehydrogenases in the lipoxygenase-hydroperoxide lyase (LOX-HPL) pathway

In terms of the metabolites of the LOX-HPL pathway, M enhanced the accumulation of *α*-linolenic acid, linoleic acid, and 13(S)-HPOT at E-L 35.5 in both vintages and in E-L 33 of 2016 (Fig. 5). However, at harvest, the difference of these precursors was weakened. Regarding the responsible genes, M had no consistent effect on the expressions of *VviLOXs* and *VviHPLs* in two vintages, however, most of them presented at higher transcription levels at harvest. C_6_ and C_9_ aldehydes can be reduced to alcohol by alcohol dehydrogenases (ADH), M upregulated most of the *VviADHs* genes at E-L 33 and 35.5 stages in 2016 and at E-L 35.5 and 38 stages in 2017 (Fig. S8).

### Identification of co-expressed transcriptional regulators and modules associated with black inter-row mulching responsive metabolites

In this study, we applied WGCNA analysis to identify the gene modules closely related to the black inter-row mulching responsive metabolites, the correlation analysis was conducted based on eigenvectors of gene modules and metabolite concentrations (Fig. S9). The absolute coefficient ≥ 0.8 and *p* value < 0.05 were defined as highly significant correlation, and the absolute coefficient ranging from 0.4 to 0.8, and *p* value < 0.05 was defined as moderate correlation. The turquoise module was highly correlated with flavonoids and had moderate correlations with sucrose, glutamate, ornithine, arginine, terpenes, and C6/C9 compounds. Brown module was highly correlated with glutamate and showed moderate correlations with ornithine, arginine, phenylalanine, ABA, and terpenes. The yellow module was highly correlated with arginine and had moderate correlations with ornithine, phenylalanine, ABA, terpenes, and C6/C9 compounds. The green module was moderately correlated with sucrose, glutamate, and flavonoids. The red module had moderate correlations with sucrose, flavonoids, and C6/C9 compounds. The black module was moderately correlated with glutamate, ornithine, arginine, and terpenes. The Magenta module had moderate correlations with glutamate, phenylalanine, ε-viniferin, ABA, and norisoprenoids. Besides correlation analysis, we summarized the distribution of differentially expressed genes involved in the interested pathways as shown in Fig. S9.

The hub genes of each module with absolute module membership greater than 0.8 and absolute kME ranking within the top 10% were selected. Those hub genes annotated as transcription factors were regarded as the key regulators to the accumulation of black inter-row mulching responsive metabolites such as flavonoids, terpenes, norisoprenoids, etc. The size and KEGG enrichment analysis for each identified module, the correlation analysis based on eigenvectors of gene modules, and metabolite concentrations were described in Text S3. Based on the above, we summarized the biological significance of each module as follows: the turquoise module was related to photosynthesis, chlorophyll metabolism, glutamate metabolism, flavonoid metabolism, terpene biosynthesis, and LOX-HPL pathway; blue module was mainly related to sucrose metabolism and flavonoid metabolism; brown, yellow, red and magenta modules had close association with glutamate metabolism, LOX-HPL pathway, the biosynthesis of heat shock proteins, and the biosynthesis of stilbenes, respectively.

**Fig. 7:**
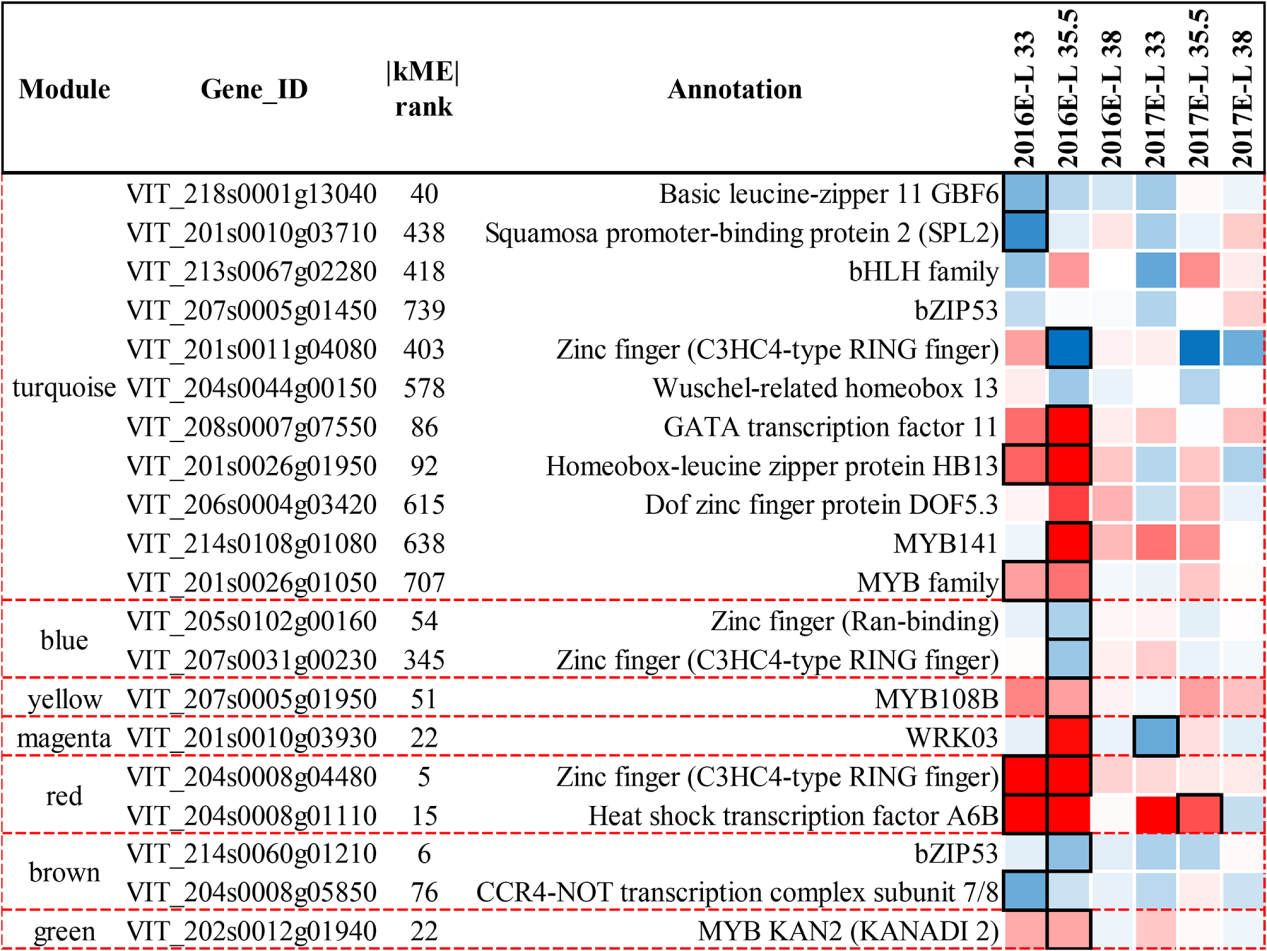
Effects of inter-row mulching on differentially expressed hub transcription factors of WGCNA modules

**Fig. 8:**
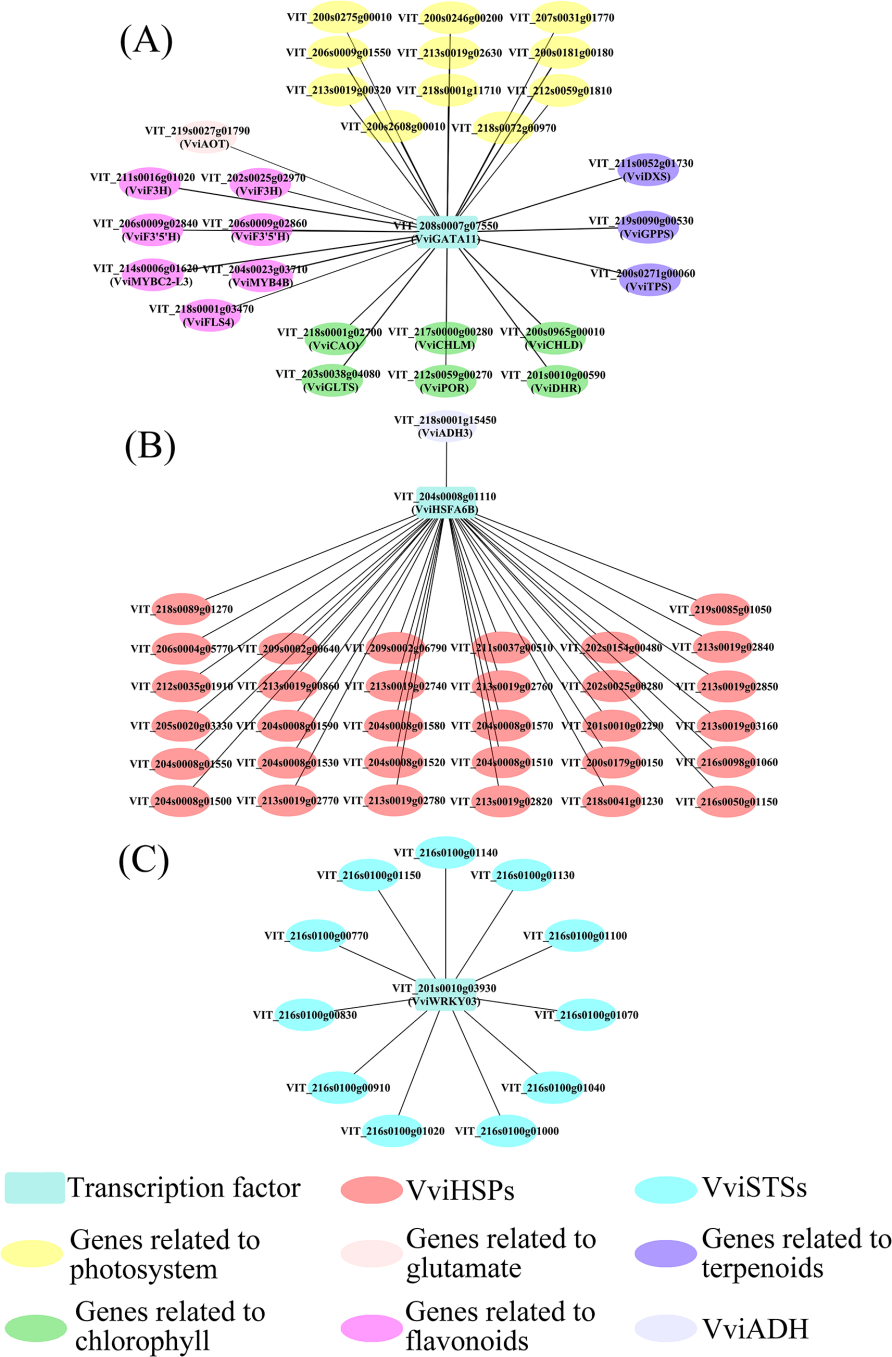
Co-expression networks of transcription factor GATA 11 (A), HSFA6B (B), and WRKY03 (C)

According to the gene connectivity within each module, we selected hub genes, especially hub transcription factors (hub TFs). In the turquoise module, eleven hub TFs were consistently affected by M in two vintages (Fig. 7), four of them annotated as bZIP11 (VIT_218s0001g13040), SPL2 (VIT_201s0010g03710), and bZIP53 (VIT_207s0005g01450) were downregulated by M at E-L 33 stage, another four hub TFs annotated as Homeobox-leucien zipper protein HB13 (VIT_201s0026g01950), DOF5.3 (VIT_206s0004g03420), MYB141 (VIT_214s0108g01080) were upregulated by M at veraison, and GATA11 was upregulated by M at E-L 33 and 35.5 stages. In the turquoise module, the DEGs co-expressed with *VvibZIP11* and *VvibZIP53* included *VviAOT* (VIT_219s0027g01790), Besides, the co-expressed DEGs with *VviGATA11* included genes involved in chlorophyll biosynthesis such as *VviGLTS*, *VviCHLD*, *VviCHLM* (Fig. 8).

In the red module, 2 hub TFs, annotated as zinc finger family transcription factor (VIT_204s0008g04480) and heat shock transcription factor HSFA6B (VIT_204s0008g01110) respectively, were identified as hub TFs, and they were downregulated by M at E-L 33 and 35.5 stages (Fig. 7). In addition, a total of 36 heat shock protein genes were included in the red module, and they were highly co-expressed with HSFA6B. It was noted that *VviADH3* (VIT_218g0001g15450) involved in the LOX-HPL pathway was included red module, and it was co-expressed with *VviHSF6B* (Fig. 8).

In the magenta module, 15 out of 33 genes were annotated as stilbene synthase genes (*VviSTSs*). Besides, the eigenvector of the magenta module was moderately correlated with *ε*-viniferin concentrations. Two hub TFs, annotated as zinc finger family transcription factor (VIT_2120028g02530) and WRKY03 (VIT_201s0010g0390) respectively, were identified in the magenta module (Fig. 7), and were upregulated at E-L 33 stages and were downregulated by M at veraison.

## Discussion

### The transcriptomic profiles and DEGs

Under the same screening condition, there were far more DEGs in 2016 than in 2017, this confirmed that the influence of inter-row mulching on gene transcription levels was highly dependent on vintage. In the present study, vintage 2017 was characterized as stronger light compared to vintage 2016 [4], so the cluster-zone light changes caused by inter-row mulching might had less impact on the gene expression of grapes in 2017 and thus resulted in fewer DEGs in 2017.

Under high temperatures, heat shock transcription factors (HSFs) are recognized as the principal regulator of the heat shock response. In this study, M upregulated the expression of 37 genes encoding HSPs, and 9 genes encoding HSFs were also significantly regulated, supporting that M grapes were subjected to heat stress (Fig. 1). The results of the correlation analysis were consistent with previous studies that *VviHSF01*, *VviHSF05*, *VviHSF15*, and *VviHSF18* may be key transcription factors that regulate *VviHSP* expression under high-temperature stress conditions.

Among the twelve down-regulated genes at E-L 33 stage, transcription factor TSRF1 (VIT_207s005g03230) could respond to ethylene and bind to the GCC box in the promoters of genes related to pathogens and diseases, and further induce these genes’ expression [16]. In addition, TSRF1 could increase the tolerance of rice to osmotic stress and drought stress [17]. Previous study has confirmed that inter-row mulching decreased the evaporation of soil water [2]. Besides, M grapes were more easily subjected to drought stress compared to C grapes, which could result in higher expressions of *VviTSRF1* in M grapes than in C grapes. Whether lower ABA levels in M grapes than in C grapes were related to the downregulation of *VviTSRF1* in M grapes needs to be further studied.

At veraison, genes related to photosynthesis and genes encoding NADPH were upregulated, besides, the expression of *VviFECH* (VIT_207s0031g03200 and VIT_204s0008g00800) in 2016 veraison, and *VviCLH* (VIT_207s0151g00130,) in 2016 E-L 33 was downregulated by M. These results suggested that the more protoporphyrin IX shift to the Mg branch of chlorophyll synthesis, and the degradation of chlorophyll was inhibited. Overall, our results proved that M grapes promoted chlorophyll biosynthesis under more abundant chlorophyll precursors, whereas inhibited the degradation of chlorophyll at E-L 33 and 35.5 stages, which could lead to higher levels of chlorophyll in M grapes than in C grapes.

In summary, the transcriptomic profiles suggested that black inter-row mulching treatment promoted the photosynthesis of grape berries as a compensatory effect after light reduction and suffered heat stress. The biological responses of C grapes revealed the growing conditions of winegrapes in northwest regions and consistently modifying multiple metabolic pathways in grape berries.

### Primary metabolites and transcription pathway

The level of sugar and organic acids strongly correlated with the ripening process of grape berries. The efflux efficiency of sucrose reflected photosynthesis rate, and the attenuated solar reflection caused by M led to decreases in photosynthesis rate and further resulted in less accumulation of photo assimilate such as sucrose. This indicated that the up-regulated chlorophyll synthesis did not reverse the decrease in photo assimilate due to the attenuated light, which in turn led to lower concentrations of fructose and glucose at E-L 33, as well as retarded the accumulation of organic acids (Table 1). Higher temperatures observed in M at veraison were beneficial for the sugar accumulation process, however, the lower concentration of TCA intermediates, such as aconitate, fumaric acid, and succinic acid, indicated inhibition of the TCA cycle and resulted in lower organic acids at E-L 35.5. At harvest, the lower sugar and higher acids suggested that M grapes were less ripe than C grapes. Although the elevated temperatures accelerated the accumulation of grape sugars in vintage with weaker light (2016), in years with adequate temperatures, the absence of light resulted in slower ripening. This implied a key role of sunlight rather than temperature in the ripening process of grape berries in semi-arid regions.

Amino acids are the main nitrogen compounds in grape berries. In this study, the underground soil under black inter-row mulching contained higher total nitrogen or efficient nitrogen concentrations than controls at different depths [6], which could favor grapevines of M to assimilate more nitrogen than controls and thus lead to more accumulation of glutamate than controls at veraison and harvest (Fig. S5). Attributed to the much greater supply of precursor (glutamate), the concentrations of glutamine, proline, ornithine, and arginine, derived from the glutamate metabolism pathway, were higher in M berries than in C berries, which were also confirmed by the upregulation of *VviGS*, *VviAOT*, and *VviASSY* in M berries (Fig. 2). In terms of aromatic amino acids, chorismate mutase (CM) controls the flow direction of chorismic acid to the biosynthesis branch of phenylalanine or tyrosine. The upregulation of *VviSDH* and *VviSK* caused by M could also result in more accumulation of chorismic acid in M berries, and consequently lead to a higher concentration of phenylalanine in M berries than in C berries, albeit the downregulation of *VviCM* by M.

### Phenolic metabolites in phenylpropanoid metabolism

Based on the metabolome analysis, the stilbenoid *ε*-viniferin was identified as the sensitive metabolite in response to M treatment. To be specific, M decreased *ε*-viniferin concentrations at E-L 33 stage in 2016 and 2017, and at E-L 35.5 stage in 2017, whereas increased its concentrations at E-L 35.5 stage in 2016, which was consistent with the influence of M on *VviSTSs* expressions as mentioned above (Fig. 4). These results confirmed that *VviSTS* played a key role in stilbenes biosynthesis. Therefore, we attributed the lower concentrations of *ε*-viniferin in M grapes than in controls to the downregulation of M on *VviSTS* expressions.

Phenylalanine lyase (PAL) is an entry enzyme of phenylpropanoid metabolism, controlling the conversion of phenylalanine to *trans*-cinnamic acid. It was noted that the influence of M on the expressions of 2 *VviPAL* genes (VIT_211s0016g01520, VIT_211s0016g01660) was in parallel with the differences in phenylalanine concentrations between M and C berries (Fig. 4, Fig. S5). In grapes, five flavonol synthase genes (*VviFLS1-5*) have been identified, previous studies reported that the expressions of *VviFLS2*, *VviFLS4*, and *VviFLS5* showed downward trends before veraison [18]. The expressions of *VviFLS4* and *VviFLS5* drastically increased in parallel with the accumulation of flavonols during ripening, suggesting these two genes played key roles in flavonol biosynthesis in berries in the current study. Besides, previous studies revealed that light exposure affected flavonol concentrations in grapes mainly by regulating *VviFLS4* expressions [18]. In this study, M significantly downregulated *VviFLS4* (VIT_218s0001g03470) expression at veraison in 2016, whereas showed an up-regulation tendency in 2017 despite no statistical significance. GT5 (uridine diphosphate (UDP)-glucuronic acid: flavonol-3-*O*-glucuronosyltransferase) and GT6 (bifunctional UDP-glucose/UDP-galactose: flavonol-3-*O*-glucosyltransferase/galactosyltransferase) have been identified as glycoside transferase genes (GT5 and GT6) in grapes which are responsible for the glycosylation of flavonols [19]. In the current study, the downregulation of *VviGT6* maybe responsible for the decrease of flavonols in M grapes.

Flavonoids are primarily synthesized in the cytoplasm and then transported to vacuoles. As for the transport and trafficking of flavonoids, the putative mechanisms include vesicle trafficking, tonoplast transport, and the involvement of glutathione *S*-transferase (GST) in the vacuolar sequestration of anthocyanins [20]. Previous studies confirmed that Multidrug And Toxic Extrusion (MATE) transporters are responsible for the vacuolar transport of acylated anthocyanins in grapes [21]. In this study, M significantly downregulated a gene encoding MATE2 transporters especially at veraison in 2016, besides, the expression of this gene in M grapes was also moderately lower than in C grapes at E-L 35.5 in 2017 (Fig. 4). Tonoplast transport of anthocyanins can also be mediated by ATP-binding cassette (ABC) transporters. Previous studies suggested that an ABC protein, ABCC1, transports malvidin 3-*O*-glucoside, and *ABCC1* is expressed in exocarp throughout berry ripening with a significant increase at veraison [22]. In this study, M significantly downregulated *VviABCC1* (VIT_216s0050g02480) at veraison in 2016, and it showed similar effects in 2017. In grapes, five GST genes (*VviGST1-5*) have been identified. A previous study found that *VviGST1* and *VviGST4* were positively correlated with anthocyanin accumulation [23]. Another study demonstrated that *VviGST3* played a key role in proanthocyanin accumulation, *VviGST4* was relevant to the transport of anthocyanins in skins and proanthocyanidin in skins and seeds, *VviGST1* was involved in the transport of proanthocyanidin and/or flavonols in green berries [24]. In this study, the expressions of *VviGST1* were quite low, especially at veraison and harvest, the expressions of *VviGST2* were relatively high and peaked at E-L 33 stage. According to the dynamic changes of flavonoids in grapes, we speculated that *VviGST2* might play a key role in the accumulation of proanthocyanidin and flavonols. However, M did not significantly affect the expressions of *VviGST2* at E-L 33 stage. The expressions of *VviGST4* and *VviGST5* in grapes drastically increased at veraison (FPKM range in 300–600), and slightly declined at harvest, which was consistent with the changes of anthocyanins concentration. Also, the lower expression of *VviGST5* in M grapes was consistent with anthocyanin concentrations. So, our results showed that GST5 in grapes might be involved in the transport and accumulation of anthocyanin, and its function needed to be further studied. To sum up, M downregulated several key genes involved in flavonoid metabolism in 2016, including *VviF3’5’H*, *VviGT6*, *VviFLS4*, *VviUFGT*, *VviAOMT*, *VviMATE2*, *VviABCC1*, which could result in less anthocyanins accumulated in grapes. However, in 2017, a vintage with strong light, M only moderately downregulated *VviGT6*, *VviUFGT*, *VviMATE2*, and *VviABCC1* in 2017, which indicated that the expression of flavonoid biosynthesis is more likely to be light-driven.

The transcription factors involved in the phenylpropanoid pathway have been widely studied. As for the regulation of stilbenes biosynthesis in grapevines, MYB14 and MYB15 have been demonstrated to specifically activate the promoters of STS genes, *VviWRKY24* could function as a singular effector to activate the *VviSTS29* promoter, *VviWRKY03* could act through a combinatorial effect with *VviMYB14* [25–27]. In this study, M significantly upregulated the expressions of *VviMYB14*, *VviMYB15*, and *VviWRKY03* at veraison in 2016, which was consistent with the upregulation of *VviSTS* in M berries (Fig. 4). As for the regulation of flavonoid metabolism, MYBPA1, and MYBPA2 can regulate several key genes in the upstream phenylpropanoid pathway such as *VviCHS* and *VviCHI*, and the key genes involved in biosynthesis of proanthocyanidin such as *VviLAR* [14]. This presented consistency with the expression pattern in our study. As for the regulation of flavonol biosynthesis, MYBF1 can regulate the expressions of *VviFLS4* [28], it was considered that weak light would downregulate the expressions of *VviHY5* and *VviMYBF1*, and then repress flavonol biosynthesis [29]. Unexpectedly, only *VviHY5* was down-regulated at E-L 33 in 2016, and *VviMYBF1* did not present to be down-regulated by M. A possible reason for the result may be the limited attenuation of reflected light caused by M, and in particular, UV-B was not significantly attenuated (data unpublished). This result indicated again that the lower flavonols was not driven by the expression of FLS or MYB transcription factors predominantly, but by *VviGT6* in the current study. It was noteworthy that M upregulated the expressions of *VviMYB4A*, *VviMYB4B*, *VviMYBC2-L1*, and *VviMYBC2-L3* at veraison in two vintages, which was closely associated with the higher anthocyanin concentrations in M grapes than in C grapes. This indicated the key role of these transcription factors in regulating flavonoid biosynthesis, and worthy further study.

### Isoprene and metabolism and LOX-HPL pathway

In this study, (*E*,*E*)-geranyl linalool concentrations were lower in M grapes at most stages (Fig. 5). Similarly, our previous reports based on GC-MS analysis revealed that M also inhibited the accumulation of monoterpenes such as geraniol. *β*-Carotene is the precursor of (*E*)- *β*-damascenone, so its lower levels in M grapes than in C grapes in 2016 could explain the negative influence of M on (*E*)- *β*-damascenone from the substrate level. Since xanthoxin is the precursor of abscisic acid (ABA), the decreases of xanthoxin levels caused by M at E-L 33 stage could lead to reductions in ABA levels in grapes at veraison (Fig. 5). It has been demonstrated that ABA can promote grape ripening and flavonoid biosynthesis [30]. Therefore, the inhibition of flavonoids in grapes caused by M might be relevant to the lower levels of ABA in M grapes at veraison.

Terpenes in plants are derived from the cytosol-localized mevalonate pathway (MVA) and the plastid-localized 2-C-methyl-d-erythritol-4-phosphate (MEP) pathway (Fig. 6). Monoterpenes are biosynthesized in the MEP pathway, the higher level of 1-deoxy-d-xylulose-5-phosphate presented consistency with the level of *VviDXS* gene (VIT_205s0020g02130). *VviTPSs* belonging to the *TPS-b* and *TPS-g* subfamilies have been characterized as monoterpene synthases, while most of the *VviTPS*-*a* genes were involved in the sesquiterpene biosynthesis [31]. The downregulation of these TPS and DXR could lead to fewer terpenes accumulated in M grapes (Fig. 6). Besides, a large fraction of terpenoids are present as nonvolatile terpene glycosides, and three glycosyltransferases (GT7, GT14, and GT15) contributing to the production of terpene glycosides have been functionally characterized [32, 33]. The downregulation of *VviGT14* and *VviGT15* could result in a decrease in terpene glycosides in M grapes (Fig. 6).

Geranylgeranyl diphosphate (GGPP) derived from the MEP pathway is the immediate precursor of carotenoids and diterpenes. In this study, M showed a tendency to upregulate *VviGGPPS* (VIT_205s0020g01240) at three developmental stages in 2016 and 2017 (Fig. S6), which was consistent with the significantly higher level of GGPP at E-L 35.5 (Fig. 5). Based on the effects of M on *VviGGPPS*, *VviPDH*, and *VviLECY*, we concluded that M tended to promote the accumulation of carotenoids in grapes. However, at the metabolite level, M only increased *β*-carotene concentrations in 2017, this may be related to more abundant light in 2017 (Fig. 5).

NCED is a rate-limiting enzyme in ABA metabolism, catalyzing the conversion of neoxanthin to xanthoxin. Thus, the downregulation of 2 *VviNCED*s explained the decrease of ABA level in M grapes. However, 3 *VviNCED*s were upregulated in 2017, which was inconsistent with the lower levels of ABA in M grapes than in C grapes, indicating that ABA biosynthesis might be regulated by other genes besides *VviNCED*. In grapes, three genes encoding CCD (*VviCCD1*, *VviCCD4a*, *VviCCD4b*) have been functionally identified [34]. The accumulation of norisoprenoids starts at veraison and shows a good parallel with the drastic upregulation of *VviCCDs*. Previous studies reported that the expression of *VviCCD1* peaked at veraison while the highest expression of *VviCCD4a* and *VviCCD4b* showed a decline during ripening [9, 34], which was consistent with the expression profiles of *VviCCDs* in this study.

ABA as a plant hormone, is a key endogenous messenger in responses to abiotic stress. In the absence of ABA, a type 2 C protein (PP2C) can interact and inhibit the kinase SnRK2 via physical interaction and its phosphatase activity (Fig. S7). In the presence of ABA, it can bind to the ABA receptor PYR/PYL/RCAR, which disrupts the interaction between the PP2C protein and SnRK2. The released active SnRK2 is then free to activate the downstream transcription factors, ABA-responsive element Binding Factors (ABFs) which target ABA-dependent gene expression [35]. Previous studies reported that ABA-receptor transcripts could be downregulated while PP2C transcripts could be upregulated under abiotic stress or exogenous ABA treatments [36]. In the current study, the expression pattern of *VviPYR/PYL* and most *VviPP2C*s indicated that C grapes were more likely subjected to abiotic stress, which may be due to their lack of mulch protection.

C_6_ and C_9_ alcohols and aldehydes are derived from LOX-HPL pathway, in which polyunsaturated fatty acids such as *α*-linolenic and linoleic acid can be oxygenated by lipoxygenases (LOX) at specific site of either 9th (9-LOX) or 13th (13-LOX) carbon to yield corresponding hydroperoxides and undergo cleavage by hydroperoxide lyase to form C_6_ and C_9_ aldehydes and oxoacids. To date, four LOXs, two Type II 13-LOXs (*VviLOXA* and *VviLOXO*), and two 9-LOXs (*VviLOXC* and *VviLOXD*) have been identified in Sauvignon Blanc grapes, and the highest expressions of *VviLOXA* and *VviLOXO* were observed in skins and seeds respectively [37]. Consistently, *VviLOXA* had the highest expression in grape berries in this study. It was reported that *VviADH1-3* expressed in grape berries, the expressions of *VviADH1* and *VviADH3* increased from berry set stage until veraison followed by a declining trend, the expression of *VviADH2* peaked after veraison which was far higher than *VviADH1* and *VviADH3* [38]. Similar expression profiles of *VviADHs* were also observed in this study. The upregulated ADHs could explain the increases in C_6_ alcohols especially (*Z*)-3-hexenol in M grapes [4].

### WGCNA modules revealed the potential transcription factors

In the turquoise module, bZIP 11 was the highest kME hub TFs (Fig. 7), previous studies identified the functions of bZIP11, SPL2, and bZIP53 in *Arabidopsis thaliana*. To be specific, bZIP11 could be negatively regulated by sucrose, and its expression could also be affected by environmental factors such as light and circadian rhythm. Besides, bZIP11 can regulate the expressions of asparagine synthetase genes and proline dehydrogenase genes [39]. In terms of bZIP53, it can coordinate with bZIP10 and bZIP25 to regulate seed maturation [40], besides, it plays a key role in regulating proline, asparagine, and branch-amino acid metabolism [41]. Based on our results and the reports from the literature, we concluded that bZIP11 and bZIP53 might be involved in glutamate metabolism. The co-expression of *VviAOT* with bZIP allowed us to speculate that bZIP11 could negatively regulate *VviAOT* expressions. The downregulation of bZIP11 caused by M would lead to the upregulation of *VviAOT* and further result in the increases in ornithine and arginine concentrations in M grapes. In higher plants, GATA transcription factors are involved in many biological processes, including light response, nitrogen metabolism, chloroplast development, seed germination, flowering, etc [42]. . Notably, a previous study reported that the *cis*-acting elements located in the upstream 2000 bp of 19 *VviGATA* genes in grapes were light-responsive, implying that *VviGATAs* could be regulated by light exposure [43]. In addition, the overexpression of *GmGATA58* in soybeans significantly upregulated the expression of genes involved in chlorophyll biosynthesis such as *CHLH*, *CHLI*, *CHLM*, and *CHLG* [44]. This suggests that GATA11 might be a sensitive transcription factor for to the attenuated solar reflection caused by M. It may further regulate key genes responsible for chlorophyll biosynthesis, thereby affecting chlorophyll levels in grape berries, and subsequently regulating berry photosynthesis process as mentioned above (Fig. S3). We also identified DEGs co-expressed with other hub TFs besides bZIP11, bZIP53 and GATA11 (Fig. 8), and these DEGs included genes encoding subunits of light reaction center, *VviHT* in sucrose metabolism, *VviDXS*, *VviGPPS*, *VviTPS* in terpene biosynthesis, *VviF3H*, *VviF3’5 H* and *VviMYB4B* in flavonoid metabolism, *VviADH* in *LOX-HPL* pathway, the interaction mechanisms between these genes and hub TFs was worthy to be further studied.

The results of the red module highlighted the importance of HSFA6B in the adaptions of grape berries to heat stress. The prolonged high temperature (> 30℃) duration caused by M could upregulate *VviHSF6B* and further induce the expressions of *VviHSPs* to protect grapes from heat damage. Based on the above, we speculated that the expressions of *VviADH3* could be regulated by that zinc finger transcription factor in response to heat stress, and consequently resulted in increases in C_6_ alcohols such as (*Z*)-3-hexenol. The regulatory mechanisms for this response need to be further assessed.

The *VviSTS*s were moderately correlated with *ε*-viniferin concentrations, which implied that the decreases of *ε*-viniferin in M grapes mainly resulted from the downregulation of *VviSTSs* caused by M. Besides, the expression pattern of WRKY 03 was also consistent with the influence of M on the expressions of *VviSTSs*. Similarly, previous studies also reported the combinatorial regulation of *VviSTSs* by WRKY03 and MYB14 [26]. Therefore, in this study, we thought that M mainly regulates the expressions of *VviSTS* through positively regulating *VviWRKY03* (Fig. 8), further affecting the accumulation of *ε*-viniferin in grapes.

To sum up, WGCNA allowed us to identify 11 hub TFs being regulated by M in the turquoise module (Fig. 7), which might regulate the expressions of *VviGLTs*, *VviCHLD*, *VviCAO* and *VviDHR* in chlorophyll metabolism, *VviHTs* in sucrose metabolism, *VviDXS*, *VviGPPS*, *VviTPS* in terpene biosynthesis, *VviF3H*, *VviF3’5’H*, *VviMYB4B* in flavonoid metabolism, to further affect the accumulation of chlorophyll, sucrose, flavonoids, terpenes, and C_6_ alcohols. Notably, we found that M could affect the expressions of *VvibZIP11* to regulate *VviAOT* and further modulate the concentrations of ornithine, arginine, the regulation of *VviGLTs*, *VviCHLD*, *VviCAO* and *VviDHR* could be mediated by *VviGATA11* under M treatment. In response to the prolonged high-temperature duration caused by M, the expressions of *VviHSPs* could be induced by *VviHSFA6B* to protect grapes from heat damage. Besides, *VviHSFA6B* could also regulate the expressions of *VviADH3* to affect the biosynthesis of C6 alcohols in M grapes. The influence of M on *ε*-viniferin concentrations in grapes could be mainly attributed to the regulation of *VviSTSs* mediated by *VviWRKY03*. The specific regulation mechanism of these hub TFs to certain metabolisms needs to be further studied.

## Conclusion

In summary, this study investigated metabolic and transcriptomic responses of Cabernet Sauvignon grapes under black geotextile inter-row mulching treatment (M) in a semi-arid climate in two vintages (2016–2017). M showed tendencies to decrease glucose and fructose concentrations in grapes, and it increased concentrations of amino acids in the glutamate pathway and phenylalanine in grapes at veraison and harvest. Based on transcriptome analysis, we found that differentially expressed gene numbers between M and control grapes at pre-veraison and veraison were far higher than those at harvest. Besides, the effects of M on grape gene expressions were more evident in a vintage (2016) with weaker light. Combined metabolome and transcriptome analysis revealed that M upregulated several key genes in the chlorophyll biosynthesis pathway and light reaction process at the pre-veraison and veraison stages. M upregulated the expression of heat shock transcription factors and heat shock proteins. The upregulation of *VviSDH* and *VviSK* at veraison led to increases in phenylalanine concentrations in M grapes. The upregulation of *VviGS*, *VviAOT*, and *VviASSY* at veraison confirmed increases in glutamine, ornithine, and arginine concentrations in M grapes. The downregulation of *VviSTS* at pre-veraison and harvest, *VviGT6*, *VviUFGT*, *VviAnthMATE2*, and *VviABCC1*, resulted in decreases in *ε*-viniferin, anthocyanin, and flavonol concentrations in M grapes. The downregulation of *VviDXR* and *VviTPS* at veraison, and *VviCCD4a* at harvest, mediated the decreases in terpenoids and norisoprenoids in M grapes. M upregulated the expressions of *VviADH* at veraison and resulted in increases in (*Z*)-3-hexenol in M grapes. In addition, we conducted WGCNA analysis and identified key transcription factors that possibly regulated the biosynthesis of the above metabolites. Taken together, this study provides a comprehensive overview of metabolic and transcriptomic responses of grapes exposed to inter-row mulching treatment in a semi-arid climate. These findings could facilitate understanding the complex regulatory network of metabolites especially phenolic and aromatic compounds biosynthesis in response to microclimate changes, as well as provide theoretical foundations for winegrowers to apply inter-row mulching in semi-arid climates.

## Materials and methods

### Experiment layout and sampling

This study was conducted in a commercial vineyard (44°14′44′′N, 86°13′55′′E) located in a county in the north-west region of China. The experimental wine region was characterized by a semi-arid climate with few precipitations (< 200 mm in growing season), strong light, high average temperature, and large temperature difference between day and night in the whole growing season. The grapevines were planted in 2010 and were spaced 2.9 m × 0.8 m with an orientation of west-south/east-north (15°). The modified vertical shoot-positioned (M-VSP) spur-pruned cordon system was applied to grapevines, of which 15–18 nodes per linear meter of the row were retained.

The black geotextile mulch strip (M) with a width of 3 m was set up on the vineyard inter-row floor from pre-flowering (late May) to harvest (October) in 2016–2017. As for control groups (C), tillage was conducted at flowering and veraison to control weed growth. Treatments were applied in both inter-rows of thirty grapevine experimental units and were replicated three times. Five grapevines at each end of the experimental plot were excluded to minimize the border effect.

Grape sampling was conducted at three phenological stages on the same day, as follows: (1) E-L 33 stage (green berries), (2) E-L 35.5 stage (mid-veraison, approximately 50% of colored grapes), (3) E-L 38 stage (harvest, TSS 24–26 °Brix). Samples were taken in the morning (before 8 AM local time) on sunny days. A total of 400 berries from 20 treated grapevines were immediately frozen in liquid nitrogen and were used for the untargeted metabolome and transcriptome analysis.

### Extraction and identification of berry metabolites using UPLC-Q-TOF-MS

A sub-sample of 50 g grapes was randomly selected from each replicate and were well ground under the protection of liquid nitrogen after removing seeds. A subset of 25 mg tissue powder was weighed and transferred to a 1.5 mL tube with the addition of 800 µL pre-cooled methanol: acetonitrile: H_2_O (2:2:1, v/v/v) solution, and the tissue power was further ground in Tissue Lyser in 50 Hz for 5 min, and the extract was subsequently sonicated for 10 min in 80 Hz. After precipitation at -20 ℃ for 2 h, the extract was centrifuged at 25,000 × *g*, 4 ℃ for 20 min, then 650 µL supernatant was collected and the residues were extracted again. The pooled supernatant was lyophilized and resolved in 600 µL 10% (v/v) aqueous methanol solution, then the mixture was sonicated in 80 Hz for 10 min. Afterward, the extract was centrifuged at 25,000 × *g*, 4 ℃ for 15 min, and the supernatant was collected. Twenty µL aliquot of each sample were pooled as a quality control (QC) sample.

Grape metabolites were monitored on a Waters 2777 series UPLC coupled with a high-resolution tandem mass spectrometer Xevo G2-XS series Q-TOF (Waters Corporation, Milford, Massachusetts, USA) which consisted of an electrospray ionization (ESI) source in both positive and negative ionization modes. Specific parameters were presented in Text S4.

### Berry RNA isolation, sequencing, and data analysis

A sub-sample of 50 berries was randomly selected from each replicate for RNA extraction, and total RNA was isolated from the frozen deseeded berries using a SpectrumTM Plant Total RNA Kit (Sigma-Aldrich, Carlsbad, CA, USA) according to the manufacturer’s instructions. The RNA concentration and purity were determined using a Nanodrop 2000 spectrophotometer (Thermo Fisher Scientific Inc., Wilmington, DE, USA), and the RNA integrity was measured using an Agilent 2100 Bioanalyzer (Agilent, Santa Clara, CA, USA). A total of 36 RNA-seq libraries (2 treatments × 3 replicates × 3 developmental stages × 2 vintages) were constructed on an Illumina HiseqTM 2000 platform to yield 150-bp pair-end reads. The clean reads were aligned against the V2 version of the *V. vinifera* 12 × genome (PN40024) using the Bowtie2 software. All the read mapping rates exceeded 70% for the respective RNA-seq libraries, which indicated the quality of sequencing data was sufficient for further investigation. Gene expression levels were normalized by determining the fragments per kilobase of transcript per million fragments mapped (FPKM) using RSEM software. The RNA-seq data has been deposited into the Sequence Read Archive (SRA) at NCBI (National Center for Biotechnology Information) with the accession number PRJNA722600.

### Quantitative real-time PCR

Six candidate genes were selected for validation of transcript quantification of RNA-seq data by quantitative real-time PCR (qRT-PCR). Firstly, total RNA isolation was the same as the procedure mentioned above. A total of 1 µg RNA was used for reverse transcription reaction using a HiScript IIQ RT SuperMix for qPCR + gDNA wiper kit (Vazyme, China) following the manufacturer’s instructions. qRT-PCR was performed using a ChamQ Universal SYBR qPCR Master Mix kit (Vazyme, China) in a Bio-Rad CFX96 Real-Time PCR system (Bio-Rad, USA). To be specific, each qRT-PCR reaction (20 µL) contains 2 µL of cDNA template, 0.4 µL of 10 mM forward primer, 0.4 µL of 10 mM reverse primer, 7.2 µL of ddH2O, and 10 µL of 2 × ChamQ Universal SYBR qPCR Mix solution. The cycling conditions were as follows: 95 ℃ for 30 s, followed by 40 cycles of 95 ℃ for 10 s, 60 ℃ for 30 s. The gene-specific primers used for qRT-PCR were listed in Table S1, and *VviUbiquitin* 1 was applied as the reference gene.

### Statistical analysis

A one-way analysis of variance (ANOVA) was conducted using “agricolae” package in R platform (version 3.6.1) to evaluate the statistical differences of metabolites between different treatments employing student’s *t*-test at *p* < 0.05. Principal component analysis (PCA) and orthogonal projection to latent structures discriminant analysis (OPLS-DA) were performed using SIMCA 14.1 (Umetrics, Umea, Sweden). Differentially expressed genes (DEGs) were identified using “DEseq2” package in R, the criteria of fold change ≥ 1.5 and false discovery rate (FDR) ≤ 0.01 were set as the thresholds for significant differential expression. Weighted gene co-expression network analysis (WGCNA) was conducted using ‘WGCNA’ package in R, and the resulting network connections between the identified hub genes and the interested DEGS were visualized by Cytoscape 3.5.1 (http://cytoscape.org) using a circular layout.

### Electronic supplementary material

Below is the link to the electronic supplementary material.

Supplementary Material 1

Supplementary Material 2

Supplementary Material 3

## Data Availability

Data supporting this study are included within the article and/or supporting materials. The RNA-seq data has been deposited into the Sequence Read Archive (SRA) at NCBI (National Center for Biotechnology Information) with the accession number PRJNA722600.
