# Supplementary material for: Vineyard microclimate alterations induced by black inter-row mulch through transcriptome reshaped the flavoromics of cabernet sauvignon grapes
Source: BMC Plant Biol. 2024 Apr 9;24:258. doi: 10.1186/s12870-024-04986-w (PMC11003005; doi:10.1186/s12870-024-04986-w)
Supplement: Supplementary file 1 — Supplementary Material 1 [file 12870_2024_4986_MOESM1_ESM.docx]

**Vineyard microclimate alterations induced by black inter-row mulch through transcriptome reshaped the flavoromics of Cabernet Sauvignon grapes**

Meng-Bo Tian^a,c,1^, Yu Wang^b,1^, Xiao-Tong Gao^a^ , Hao-Cheng Lu^a,c^, Qi Zhang^b^, Xiao Han^a,c^, Hui-Qing Li^a,c^, Ning Shi^a,c^, Chang-Qing Duan^a,c^, Jun Wang^a,c*^

## ^a^ Center for Viticulture and Enology, College of Food Science and Nutritional Engineering, China Agricultural University, Beijing 100083, China

## ^b^ Anhui Engineering Laboratory for Agro-products Processing, College of Tea & Food Science and Technology, Anhui Agricultural University, Hefei 230036, China

## ^c^ Key Laboratory of Viticulture and Enology, Ministry of Agriculture and Rural Affairs, Beijing 100083, China

## ^1^ These authors contributed equally to the study. ^*^Corresponding author: Jun Wang: [jun_wang@cau.edu.cn](mailto:jun_wang@cau.edu.cn)

## Text S1: The overview of transcriptomic profiles of grape berries

Approximately 8 GB clean data were obtained from each sample using RNA sequencing, with an average unique genome mapping ratio of 76.5% (Table S2). Genes with FPKM (fragments per kilobase of transcript per million mapped fragments) greater than 0.1 in at least 1 biological sample were defined as expressed genes, and we detected 28180 expressed genes in this study which were used for calculating the correlation coefficients between every two biological replicates. To validate the expression profiles obtained by RNA sequencing, qRT-PCR was conducted on six different transcripts. Linear regression analysis resulted in an r^2^ of 0.87, which indicated a very good correlation between the expression profiles assessed by RNA sequencing and the transcript abundance assessed by qRT-PCR (Fig. S1).

## Text S2: The k-means and KEGG analysis of differentially expressed genes in 2016 and 2017

In 2016, 4372 DEGs were grouped into 13 clusters, and cluster 1, 3, 6, 7, 11 and 12 were upregulated at E-L 33 and 35.5 stages (Fig. S10). Cluster 1 consisted of 347 genes, showing decreasing trend from E-L 33 to 38 stage, and were enriched in photosynthesis (mainly antenna protein) and translation process. Cluster 3 consisted of 245 genes, showing increasing trends during grape development, and were enriched in “transcription factor”, “peroxisome”, “glycolysis/gluconeogenesis”, “galactose metabolism” and “fructose and mannose metabolism”. It was noted that those genes enriched in “transcription factors” included 3 genes encoding ERF(VIT_218s0089g01030), AP2(VIT_2070031g00220), and AP2/ERF(VIT_219s0090g01080) in ethylene-mediated signaling. Previous studies found that AP2/ERF played key role in ethylene, ABA and jasmonic acid-mediated signaling for plant adaption under biotic or abiotic stress (Müller and Munné-Bosch, 2015). In this study, grapes under inter-row mulch were subjected to heat stress, which could upregulate *VviAP2/ERF* expression, and then regulate primary metabolism such as glycolysis, galactose and fructose metabolism. Cluster 6 consisted of 307 genes, peaking at E-L 35.5 stage, and were enriched in “tryptophan metabolism”, “cysteine and methionine metabolism”, “amino acid metabolism”, “glycolysis/gluconeogenesis” and “pyruvate metabolism”, etc. Cluster 7 consisted of 426 genes, showing decreasing trends during grape development, and were enriched in “transcription factors”, “photosynthesis”, “porphyrin and chlorophyll metabolism”, “phenylpropanoid biosynthesis” and “biosynthesis of other secondary metabolites”. Those genes in cluster 7 related to photosynthesis were mainly antenna protein genes, photosystem I related genes, and chlorophyll biosynthesis related genes. Intriguingly, the enriched transcription factors in cluster 7 consisted of a key light-response transcription factor GATA11 (VIT_208s0007g07550 and VIT_204s0008g03270). Previous studies found that, in grapes, most of putative *cis*-elements in the regions 2000 bp upstream of 19 *VviGATA11* genes were light responsive (Zhang *et al.*, 2018). Besides, GATA transcription factors are also involved in chlorophyll metabolism, the overexpression of *GmGATA58* significantly upregulated the expression of key genes involved in chlorophyll biosynthesis in soybean (Zhang *et al.*, 2020). Chlorophyll was the essential cofactor for PS Ⅱ-LHC Ⅱ and PS Ⅰ-LHC Ⅰ complexes functioning in photosystem. Based on the above results, we speculated that the 2 genes (VIT_208s0040g00390 and VIT_217s0000g00280) related to chlorophyll biosynthesis in cluster 7 could be regulated by GATA11. In this study, chlorophyll levels in M grapes were higher than in C grapes, which could partially explain the up-regulation of genes related to photosynthesis in grapes at E-L 33 and E-L 35.5 stages. In addition, cluster 7 consisted of 4 genes (VIT_207s0141g00690, VIT_204s0008g03400, VIT_208s0007g08580 and VIT_201s0011g03470) encoding transcription factors related to ethylene-mediated signaling., which was consist with the results showed in cluster 3. Cluster 11 consisted of 405 genes, peaking at veraison, and were significantly upregulated by M at veraison. Genes in cluster 11 were enriched in “transcription”, “RNA degradation”, “messenger RNA biogenesis”, “GTP-binding proteins” and “glycolysis/gluconeogenesis” etc. It was noted that the KEGG analysis of cluster 3, 6 and 11 all showed black inter-row mulch upregulated glycolysis process at veraison in 2016.

The genes in cluster 2, 4, 5, 8, 9, 10 and 13 were downregulated by black inter-row mulch at E-L 33 and 35.5 stages in 2016 (Fig. S11). Cluster 2 consisted of 427 genes, showing drastic decline from E-L 33 to 35.5 stage and followed by a slight upward trend. Genes in cluster 2 were enriched in “plant hormone signal transduction”, “photosynthesis”, “glycan biosynthesis and metabolism”, “circadian rhythm-plant” and “amino sugar and nucleotide sugar metabolism”. Those genes in cluster 2 related to photosynthesis consisted of *psbC* and *psbD* genes in photosystem PS II and *psaA* and *psaB* genes in photosystem PS I, and they encoded the subunit of PS II and PS I reaction center. Cluster 4 consisted of 463 genes, showing drastic upward trends from veraison to harvest, and were enriched in “starch and sucrose metabolism”, “polyketide biosynthesis proteins”, “messenger RNA biogenesis”, “phenylpropanoid biosynthesis” and “arginine and proline metabolism”, etc. Those genes in cluster 4 enriched in phenylpropanoid biosynthesis included 8 stilbene synthase genes (VIT_216s0100g01100, VIT_216s0100g01140, VIT_216s0100g01020, VIT_216s0100g01130, VIT_216s0100g01160, VIT_216s0100g01150, VIT_216s0100g01170 and VIT_216s0100g00830) and 1 resveratrol synthase gene (VIT_216s0100g01070). The significant down-regulation of stilbene synthase genes caused by M could cause the lower stilbene concentrations in M grapes than in C grapes. In addition, those genes in cluster 4 enriched in proline and arginine metabolism included the arginase gene (VIT_212s0028g01120). Arginase can catalyze the conversion of arginine to ornithine and urea. The down-regulation of arginase gene caused by M could lead to less arginine being hydrolyzed, and consequently resulted in higher concentration of arginine in M grapes than in C grapes. Cluster 5 consisted of 716 genes, showing declining trends, and were enriched in “transporter”, “plant hormone signal transduction”, “MAPK signaling pathway”, “lipid biosynthesis”, “fatty acid biosynthesis”, “glycosyltransferases”, “glycan biosynthesis and metabolism”, “glutathione metabolism” and “cysteine and methionine metabolism”. Cluster 8 consisted of 196 genes, showing declining trends, and were enriched in “ubiquitin mediated proteolysis”, “starch and sucrose metabolism”, “glycosyltransferases” and “flavonoid biosynthesis”, etc. It was noted that those genes in cluster 8 enriched in flavonoid biosynthesis included *VviCHS* (VIT_205s0136g00260) and *VviF3’5’H* (VIT_206s0009g02860、VIT_206s0009g02970、VIT_206s0009g02810 and VIT_206s0009g02805). *VviCHS* controlled the chalcone biosynthesis, and *VviF3’5’H* was related to the 3’5’-hydroxylated flavonoids biosynthesis, their down-regulation caused by M could explain the lower flavonoids concentrations in M grapes than in C grapes. Cluster 9 consisted of 175 genes, showing upward trends, and were enriched in “protein phosphatases and associated proteins”, “plant hormone signal transduction” and “glycosyltransferases”. Combining KEGG analysis results of cluster 2 and 5, we noted that M downregulated the genes expressions related to ABA, auxin, ethylene, jasmonic acid, and brassinosteroids mediated signaling. In this study, M decreased the light exposure around cluster zone and the water evaporation from vineyard soil. Besides, C grapes could be more easily subjected to light and drought stress. Therefore, C grapes might upregulate hormone signaling to adapt to the stress. Moreover, ABA levels in M grapes were higher than in C grapes, which could partially explain the down-regulation of genes expression in ABA mediation signaling. Cluster 10 and 13 consisted of 189 and 338 genes, respective, both peaking at veraison. Cluster 10 were enriched in “protein kinases”, “cytochrome P450”, “carbohydrate metabolism”, “biosynthesis of other secondary metabolites” and “amino sugar and nucleotide sugar metabolism”. Cluster 13 were enriched in “ubiquitin mediated proteolysis”, “phosphatidylinositol signaling system”, “N-glycan biosynthesis”, “membrane trafficking”, “glycerolipid metabolism” and “amino sugar and nucleotide sugar metabolism”.

In 2017, 419 DEGs were grouped into 8 clusters through *k*-means, however, KEGG enrichment analysis showed that only genes in cluster 3 and 6 enriched KEGG terms (Fig. S12). Cluster 3 consisted of 102 genes, showing drastic declining trends from E-L 33 to 35.5 stage and followed by a steady trend. M significantly downregulated the expressions of cluster 3 which were enriched in “polyketide biosynthesis proteins”, “MAPK signaling pathway”, “flavonoid biosynthesis”, “environmental adaption”, “circadian rhythm-plant” and “biosynthesis of other secondary metabolites”. Those genes in cluster 3 enriched in flavonoid biosynthesis included 10 stilbenes synthase genes (VIT_216s0100g00750, VIT_216s0100g0088, VIT_216s0100g0099, VIT_216s0100g01010, VIT_216s0100g01000, VIT_216s0100g01020, VIT_210s0042g0092, VIT_216s0100g01170, VIT_216s0100g00840 and VIT_216s0100g00830). The down-regulation of *VviSTSs* could explain the lower stilbenoids concentrations in M grapes than in C grapes, which was consistent with the impacts of M on *VviSTSs* and stilbenoids concentrations in 2016. Cluster 6 consisted of 169 DEGs, showing similar trends as cluster 3. Cluster 6 was enriched in “photosynthesis”, “photosynthesis-antenna proteins”, “MAPK signaling pathway-plant”, “glycosyltransferases” and “energy metabolism”, and was significantly upregulated by M at E-L 33 stage. It was noted that most of the genes in cluster 6 enriched in photosynthesis were annotated as antenna protein genes, which was consistent with the results shown in 2016.

Overall, the *k*-means and KEGG enrichment analysis of DEGs suggested that black inter-row mulch had the following common influence on grape genes expressions in two vintages: M significantly upregulated the expression of genes related to antenna proteins, which showed the potential to promote berry photosynthesis. M also decreased stilbenes synthase genes expression at E-L 33 stage. The decrease in berry exposure were normally considered not favoring photosynthesis. In this study, M decrease the solar radiation from vineyard floor and further decreased sunlight exposure around grape clusters, whereas M upregulated the expression of antenna protein genes and showed the potential to promote berry photosynthesis. We speculated that the up-regulation of photosynthesis related genes were closely associated with the increases in chlorophyll levels in grapes. M significantly upregulated 2 genes related to chlorophyll biosynthesis (VIT_208s0040g00390, VIT_217s0000g00280) at E-L 33 stage in 2016, and NADPH: protochlorophyllide oxidoreductase (VIT_219s0014g03160) gene at veraison in two vintages. Previous studies reported that stilbenes concentrations in plants were relatively low under normal conditions, whereas they would drastically increase in response to abiotic and biotic stresses due to their high sensitivity. Stilbenes were also characterized as marker compounds to evaluated whether plants were under stress or not. Based on above, we concluded that M attenuated the reflection of solar radiation from vineyard floor and soil water evaporation, thus C grapes experienced higher light exposure and severer drought stress compared to M grapes, which could lead to the up-regulation of stilbenes synthases genes and consequently the increases in stilbenes accumulations.

**Text S3: The WGCNA analysis procedure**

A total of 14092 transcripts were included into WGCNA analysis after filtering out the low expressed genes with average FPKM < 1. Firstly, nine modules with biological significance were identified (Fig. S13), and we subsequently conducted KEGG enrichment analysis for each module (Table S3). Turquoise module had the maximum size of 7532 genes, and the top 5 overexpressed KEGG terms included “photosynthesis proteins”, “ribosome”, “photosynthesis-antenna proteins”, “porphyrin and chlorophyll metabolism” and “transcription factors”, indicating that turquoise module was closely associated with berry photosynthesis process. Blue module consisted of 3768 genes which were enriched in “spliceosome”, “messenger RNA biogegesis”, “transcription”, “RNA transport”, “mRNA surveillance pathway”, etc. A total of 1218 genes were included in brown module, and they were enriched in “plant hormone signal transduction”, and most of genes were related to the auxin signaling pathway, such as *VviARF* (VIT_217s0000g00320, VIT_218s0089g00910, VIT_212s0035g01800). Genes in red module were enriched in the processes of “protein processing in endoplasmic reticulum”, “chaperones and folding catalysts” and “folding, sorting and degradation”, and most of genes encoding heat shock proteins (*VviHSPs*). Magenta module consisted of 33 genes, and most of them were stilbenes synthases. The genes in black module were mainly related to glycolysis/gluconeogenesis process.

## Text S4: UPLC-Q-TOF-MS/MS procedure and data analysis

Ten μL samples were injected into a ACQUITY UPLC HSS T3 column (100 mm × 2.1 mm, 1.8 μm, Waters, UK) maintained at 40 ℃. The mobile phase A and B were water and acetonitrile, respectively, both containing 0.1% (v/v) formic acid. The gradient elution program was as follows: 0-2 min, 0% B; 2-11 min, 0%-100% B; 11-13 min, 100% B; 13-15 min, 100%-0% B. The Q-TOF mass spectrometer conditions were as follows: for positive ionization mode, the capillary and sampling cone voltages were set at 3.0 kV and 40.0 V, respectively; for negative ionization mode, the capillary and sampling cone voltage were set at 2.0 kV and 40.0 V respectively. The mass spectrometry data were acquired in Centroid MES mode. The TOF mass range was from 50-1200 Da and the scan time was 0.2 s. As for MS/MS detection, all precursors were fragmented using 20-40 eV, and the scan time was 0.2 s. During the acquisition, the LE signal was acquired every 3 s to calibrate the mass accuracy. In addition, a QC sample was acquired after every ten samples to evaluate the stability of the UPLC-Q-TOF-MS. The mass spectra data was processed with Progenesis QI software (version 2.2), and the metabolites were tentatively identified by matching fragmentation patterns of compounds to Kyoto Encyclopedia of Genes and Genomes (KEGG) metabolite database.

In this study, a total of 7548 MS peaks and 3186 MS peaks were detected in positive and negative modes after filteration and curation, respectively. These MS peaks were annotated against KEGG database, and 1275 metabolites and 609 metabolites were identified in positive and negative modes (Table S4-5), respectively. Firstly, we conducted PCA analysis based on the ion intensities detected in grape samples and quality control samples (QC), which showed that QC samples were grouped together in positive and negative modes, indicating the stability of the detection. In addition, we conducted cluster analysis based on the ion intensities, which showed that the biological replicates from the same treatment were grouped together, suggesting the reliability of sampling (Fig. S14). In addition, grapes samples were firstly grouped according to the developmental stages, and then the samples of different vintages were separated within each stage except for E-L 38 stage in positive mode, indicating the metabolic processes in grapes were primarily influenced by developmental stage and vintage (Fig. S15). It was noted that the M and C grapes were separated at E-L 38 stage in positive mode, and then the samples were clustered according to vintages, indicating that M had greater influence than vintage on the concentrations of metabolites detected in positive mode (Fig. S15). To identify the marker metabolites contributing to the differences between M and C grapes, we built two OPLS-DA models based on the ion intensities in positive and negative modes respectively. However, most of the marker ions with VIPs >1 and *p* value < 0.05 were not annotated against KEGG database. After that, we extracted the metabolites involved in the interested pathways, including sucrose, hexoses, organic acids, amino acids, fatty acids, flavonoids, terpenoids, carotenoids, and explored the specific influence of M on these metabolites in two vintages.

# Supporting References

**Müller, M. and Munné-Bosch, S.** (2015) Ethylene response factors: A key regulatory hub in hormone and stress signaling. *Plant Physiology*, **169**, 32-41.

**Zhang, C.-J., Huang, Y., Xiao, Z.-Y., Yang, H.-L., Hao, Q.-N., Yuan, S.-L., Chen, H.-F., Chen, L.-M., Chen, S.-L. and Zhou, X.-N.** (2020) A GATA transcription factor from soybean (*Glycine max*) regulates chlorophyll biosynthesis and suppresses growth in the transgenic *Arabidopsis thaliana*. *Plants*, **9**, 1036.

**Zhang, Z., Ren, C., Zou, L.-M., Wang, Y., Li, S.-H. and Liang, Z.-C.** (2018) Characterization of the GATA gene family in *Vitis vinifera*: Genome-wide analysis, expression profiles, and involvement in light and phytohormone response. *Genome*, **61**, 713-723.
