# Supplementary material for: Vineyard microclimate alterations induced by black inter-row mulch through transcriptome reshaped the flavoromics of cabernet sauvignon grapes
Source: BMC Plant Biol. 2024 Apr 9;24:258. doi: 10.1186/s12870-024-04986-w (PMC11003005; doi:10.1186/s12870-024-04986-w)
Supplement: Supplementary file 3 — Supplementary Material 3 [file 12870_2024_4986_MOESM3_ESM.docx]

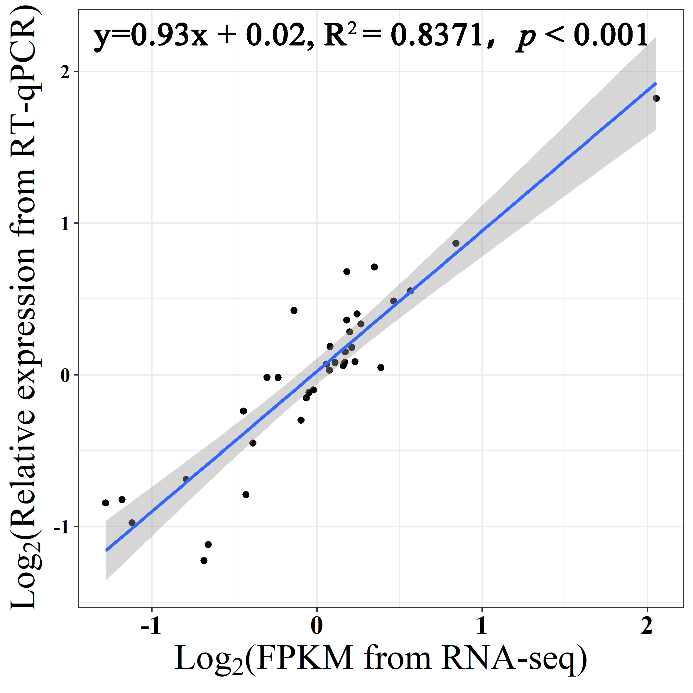
**Fig. S1** Validation of RNA-seq data by quantitative real-time PCR.


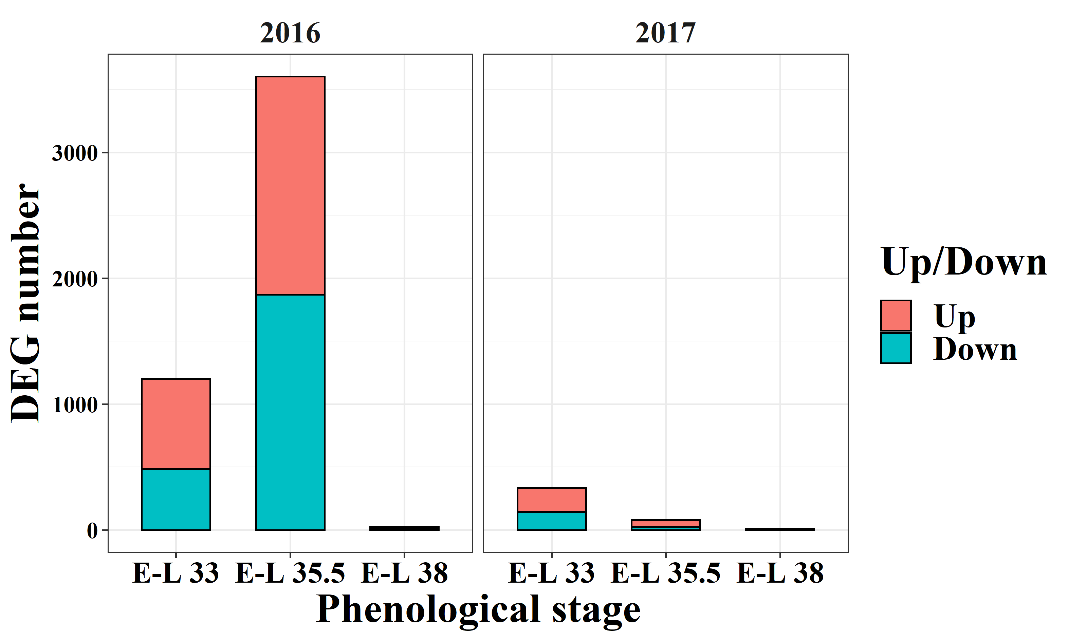
**Fig. S2** The numbers of differentially expressed genes between inter-row mulch and control grapes.

**
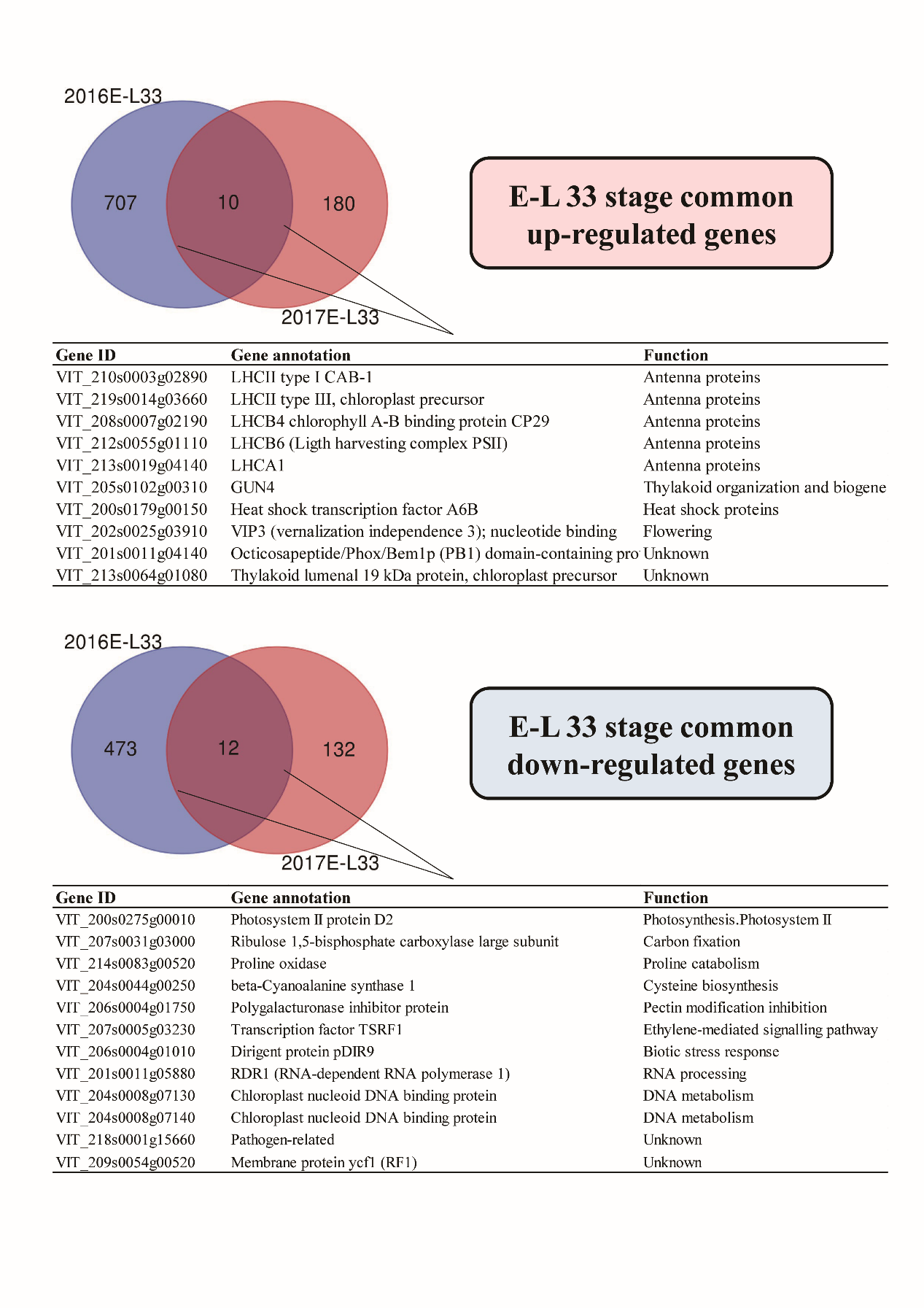
Fig. S3** Common up/down-regulated genes by inter-row mulch at E-L 33 stage in 2016-2017.


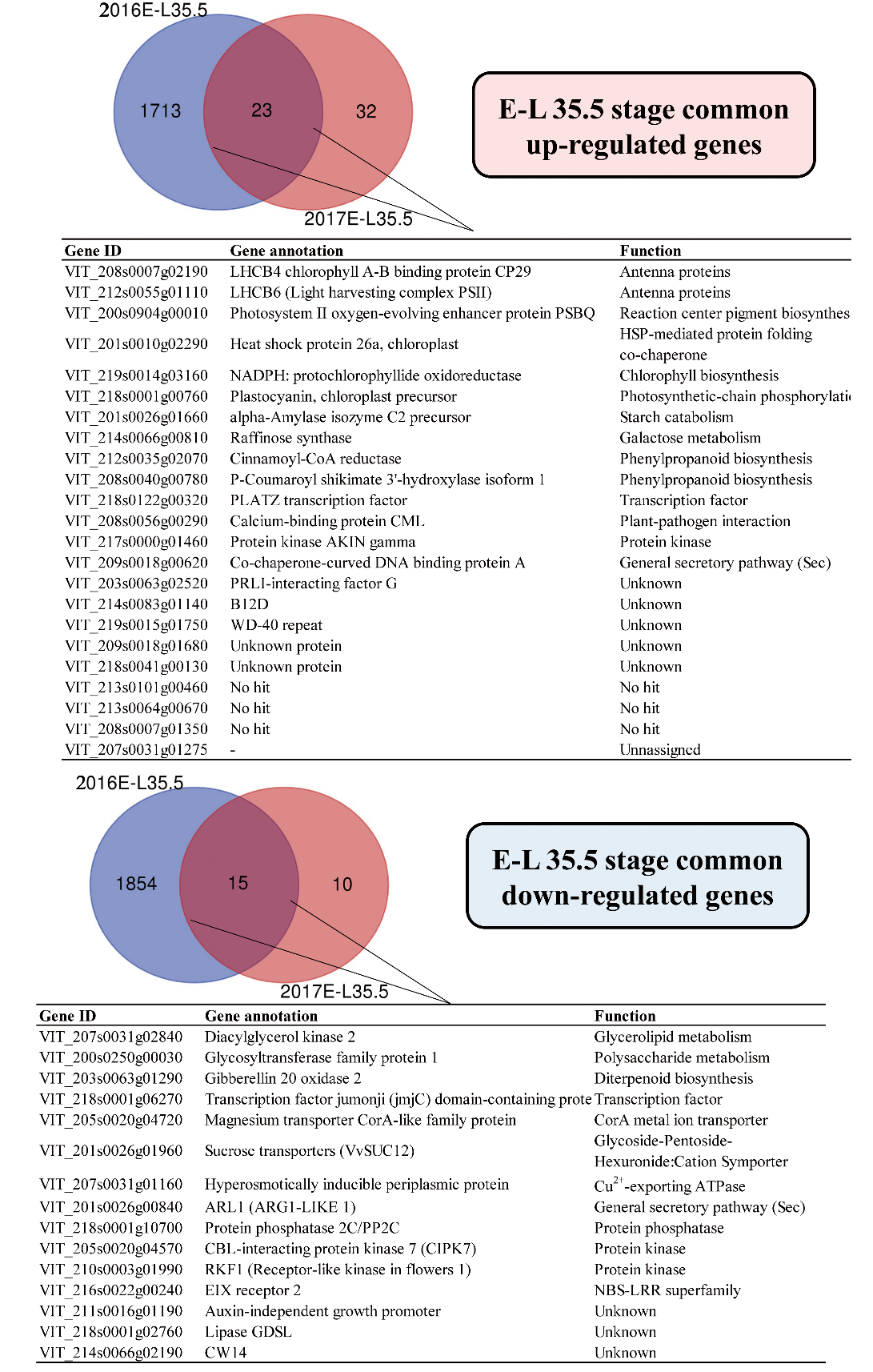
**Fig. S4** Common up/down-regulated genes by inter-row mulch at E-L 35.5 stage in 2016-2017.


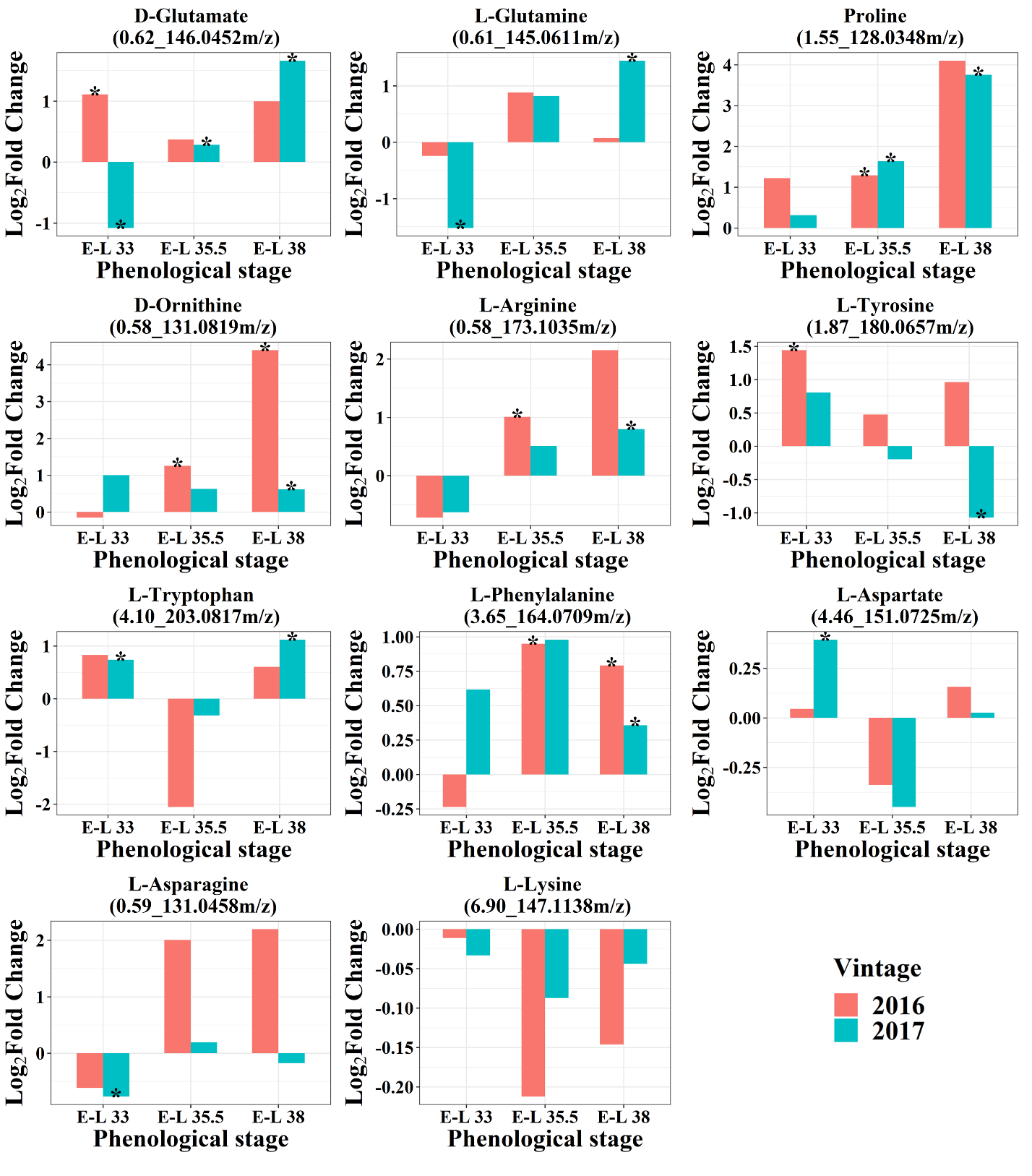
**Fig. S5** Effects of inter-row mulch on amino acids in grapes. Data represents the log_2_-transformed fold changes of metabolites ions intensities between inter-row mulch and control group. ‘*’ represents significant differences in metabolites ions intensities between inter-row mulch and control group (student’s t-test, *p* < 0.05).


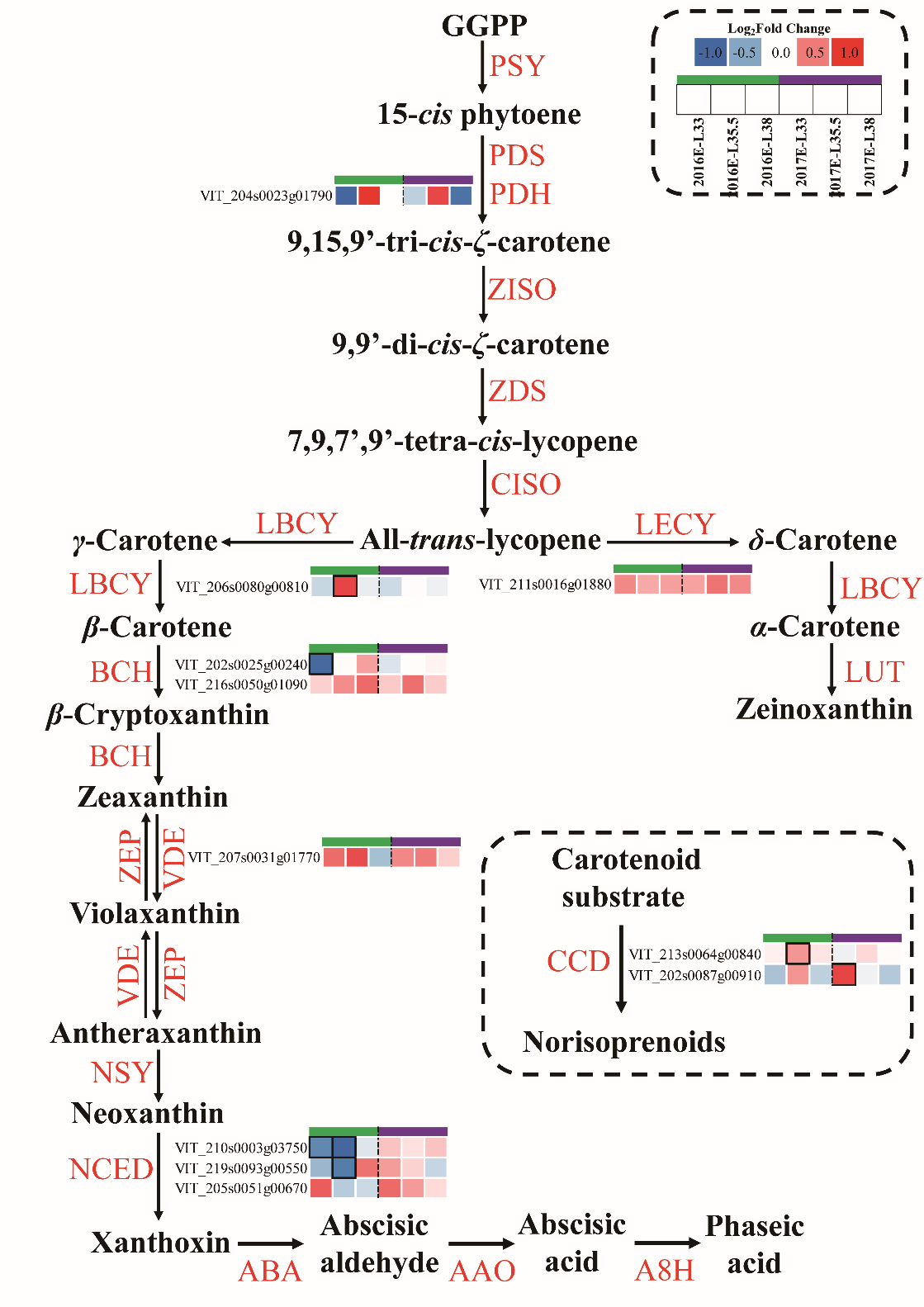
**Fig. S6** Effects inter-row mulch on expression profiles of genes related to carotenoid metabolism. Data represents the log2-transformed fold changes of gene expressions between the inter-row mulch and control group. The black border of the heatmap cell represents significant differences in gene expressions between the inter-row mulch and control group (student’s t-test, *p* < 0.05). The same as below.


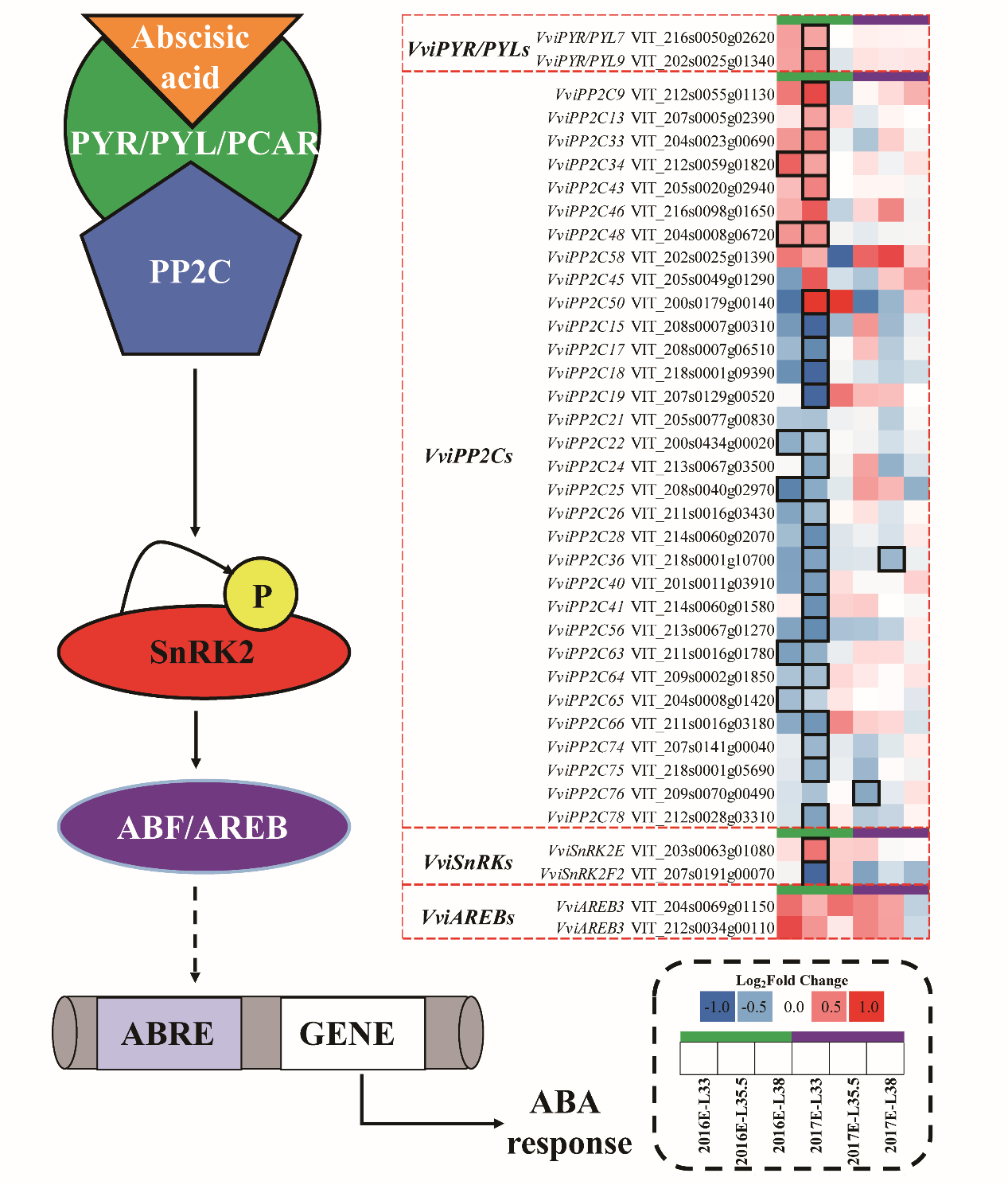
**Fig. S7** Effects of inter-row mulch on expression profiles of genes related to ABA-signaling pathway.


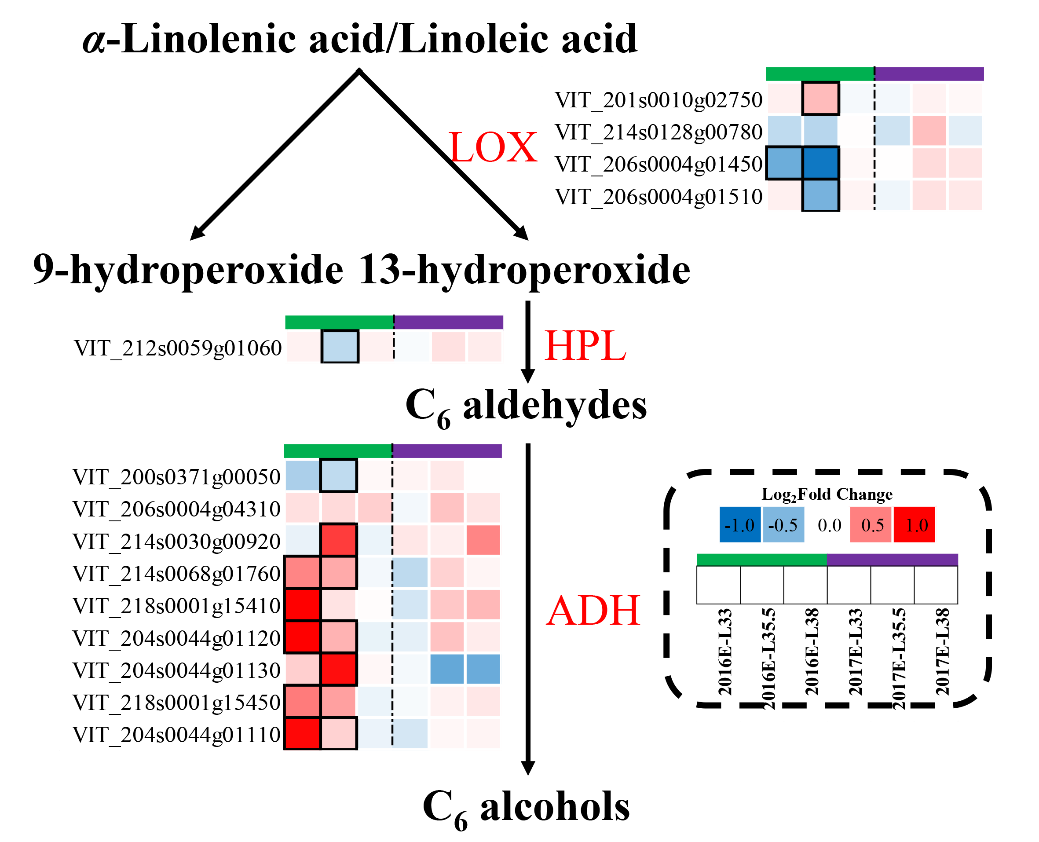
**Fig. S8** Effects of inter-row mulch on expression profiles of genes related to lipoxygenase pathway.


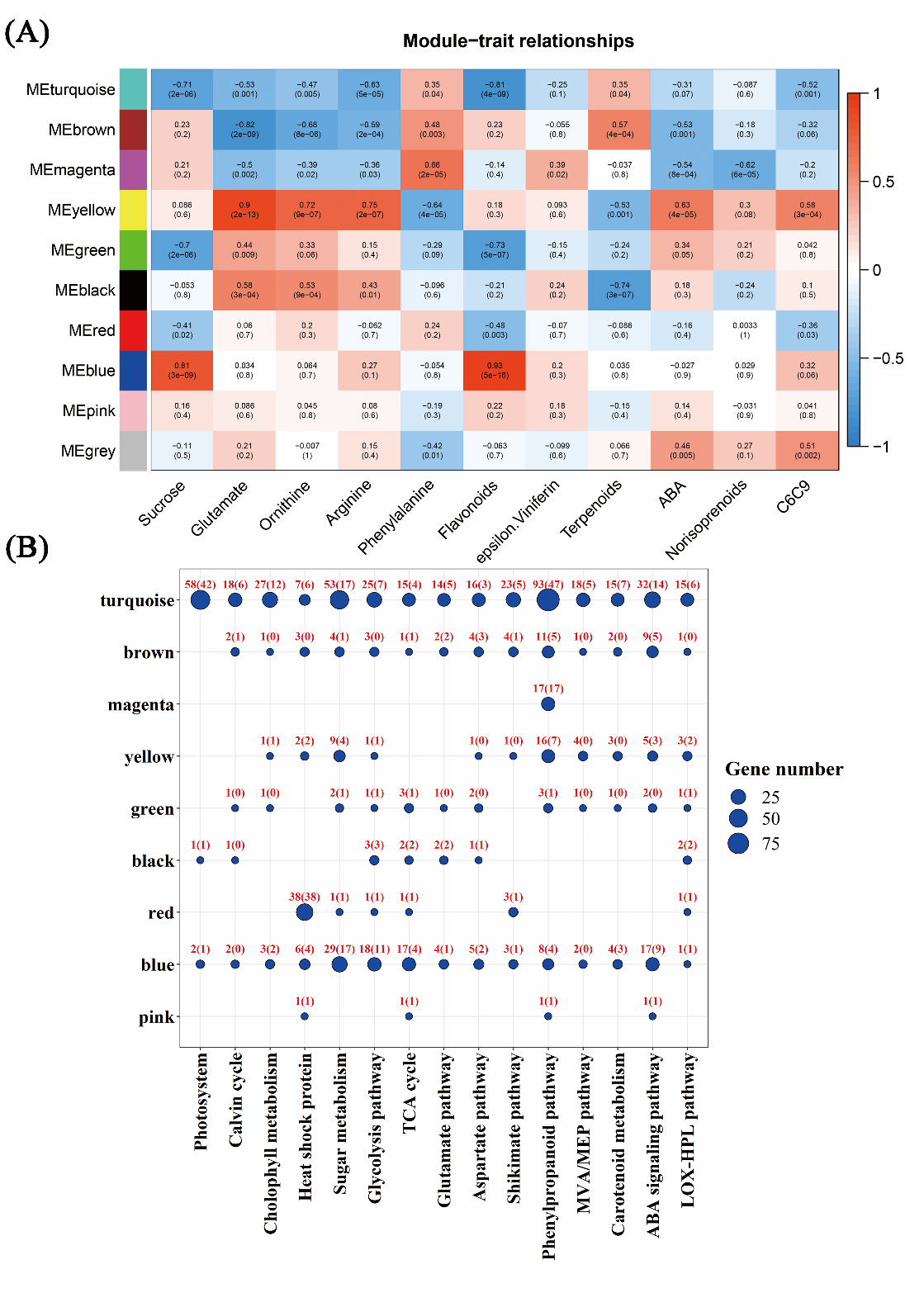
**Fig. S9** Heatmap of correlations between WGCNA module eigengenes and marker metabolites (A), and the distribution of known genes of different pathways within modules (B). Values in each heatmap cell (A) are correlations and *p*-values (in bracket), and values above each circle in the scatter plot (B) are known gene numbers and differently expressed gene numbers (in bracket) in different pathways.


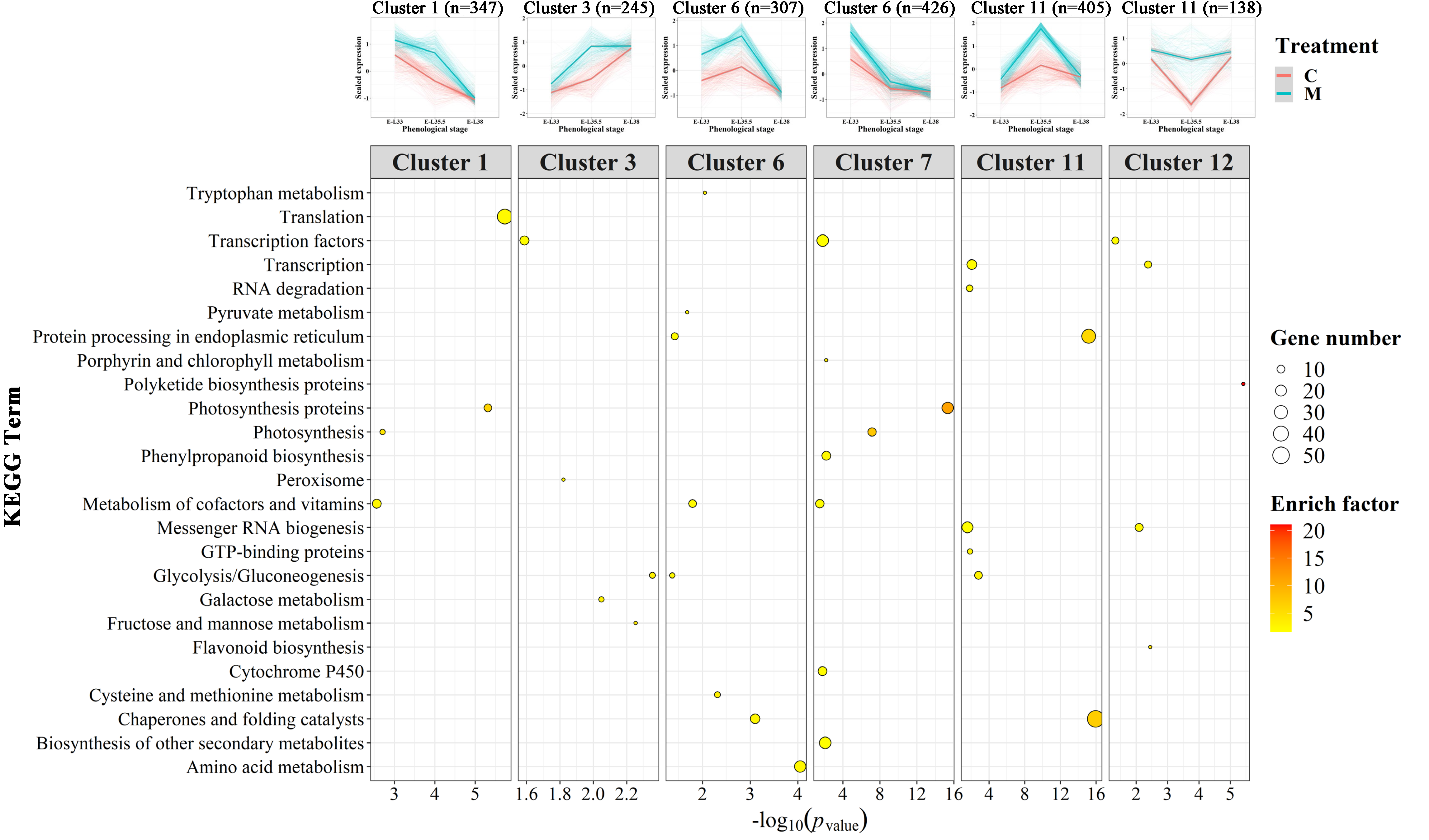
**Fig. S10** The *k*-means analysis and KEGG enrichment analysis of genes upregulated by inter-row mulch in 2016.


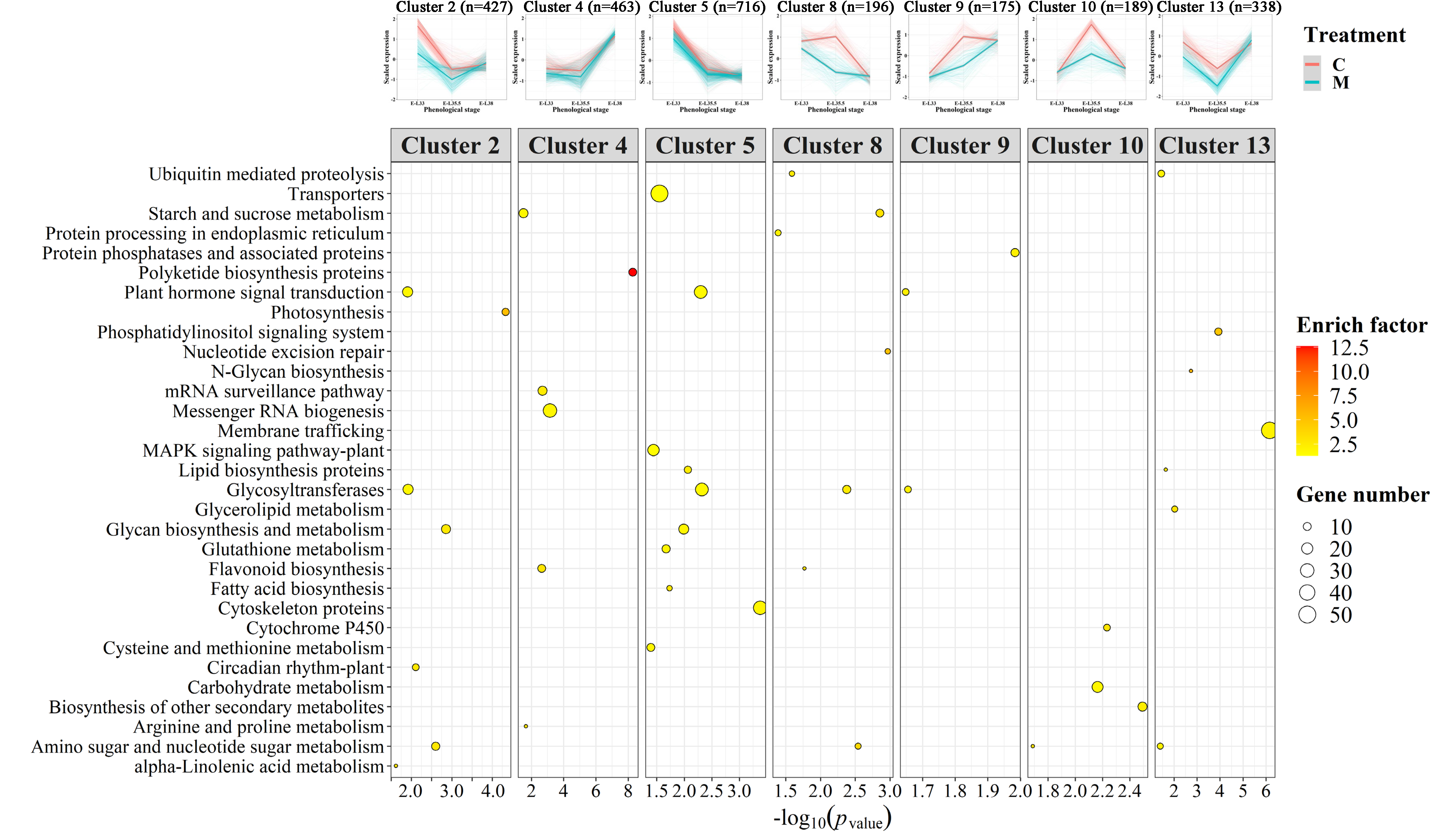
**Fig. S11** The *k*-means analysis and KEGG enrichment analysis of genes downregulated by inter-row mulch in 2016.


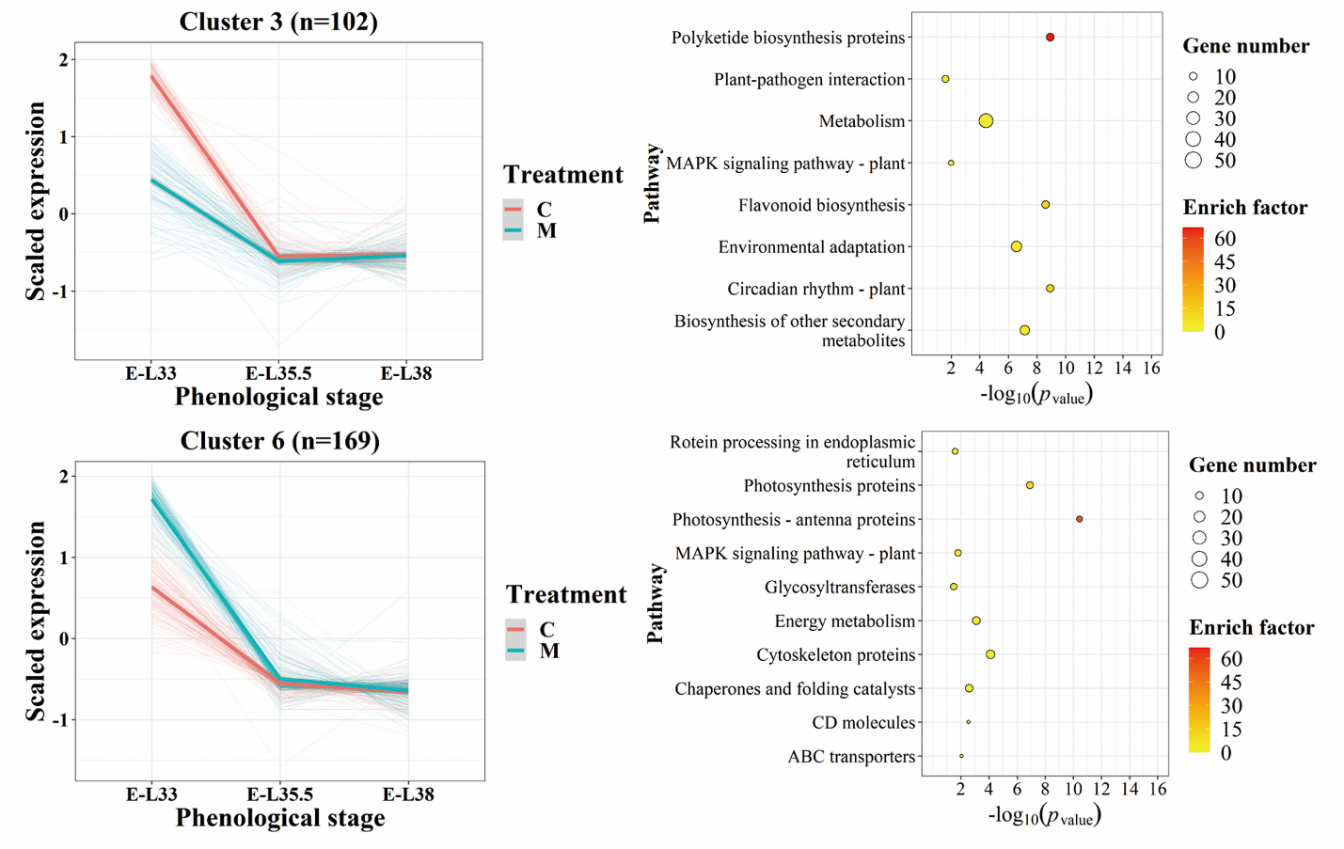
**Fig. S12** The *k*-means analysis and KEGG enrichment analysis of genes significantly regulated by inter-row mulch in 2017.


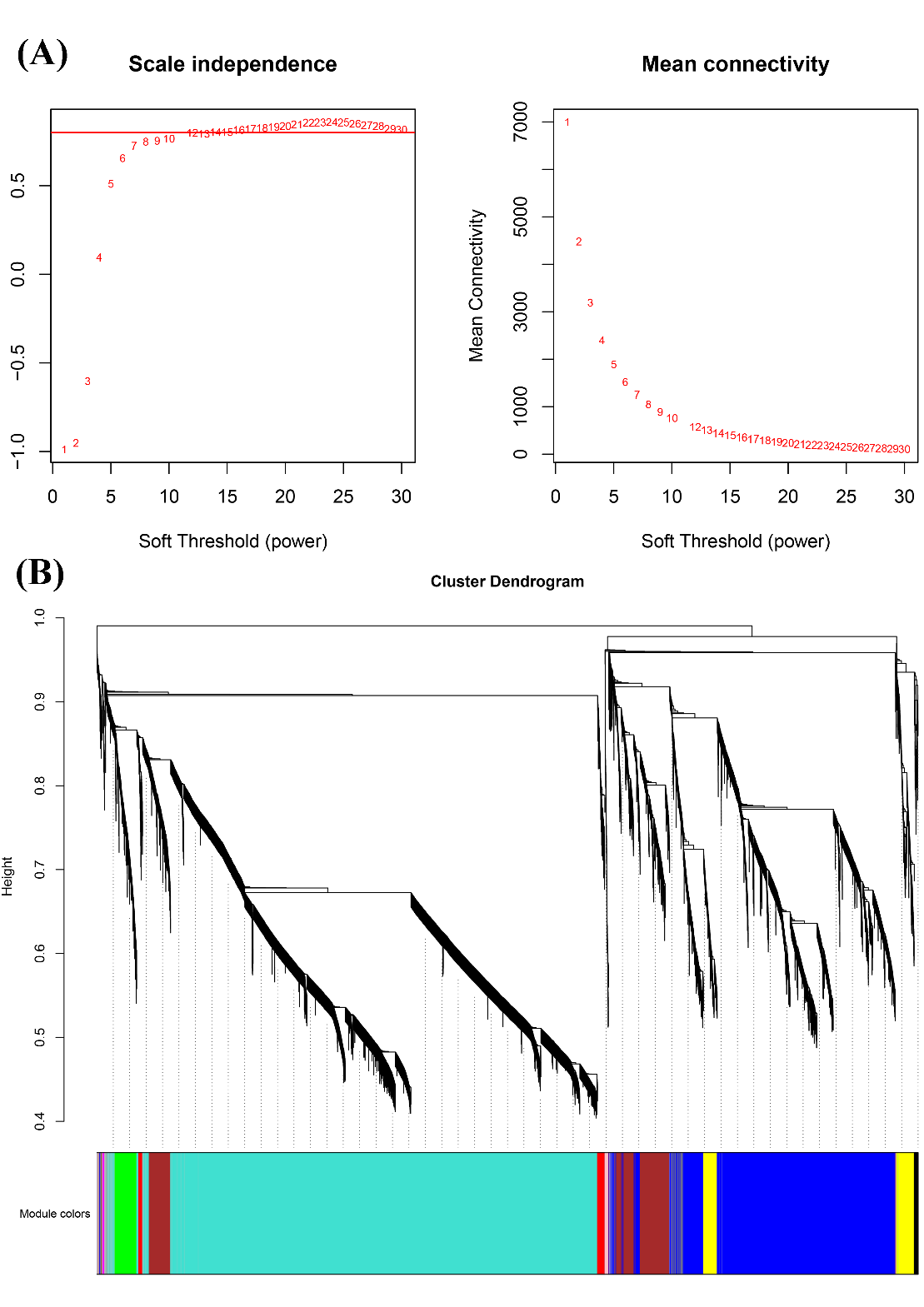
**Fig. S13** Picking soft threshold of the unscaled network (A) and identifying modules (B) in WGCNA analysis.


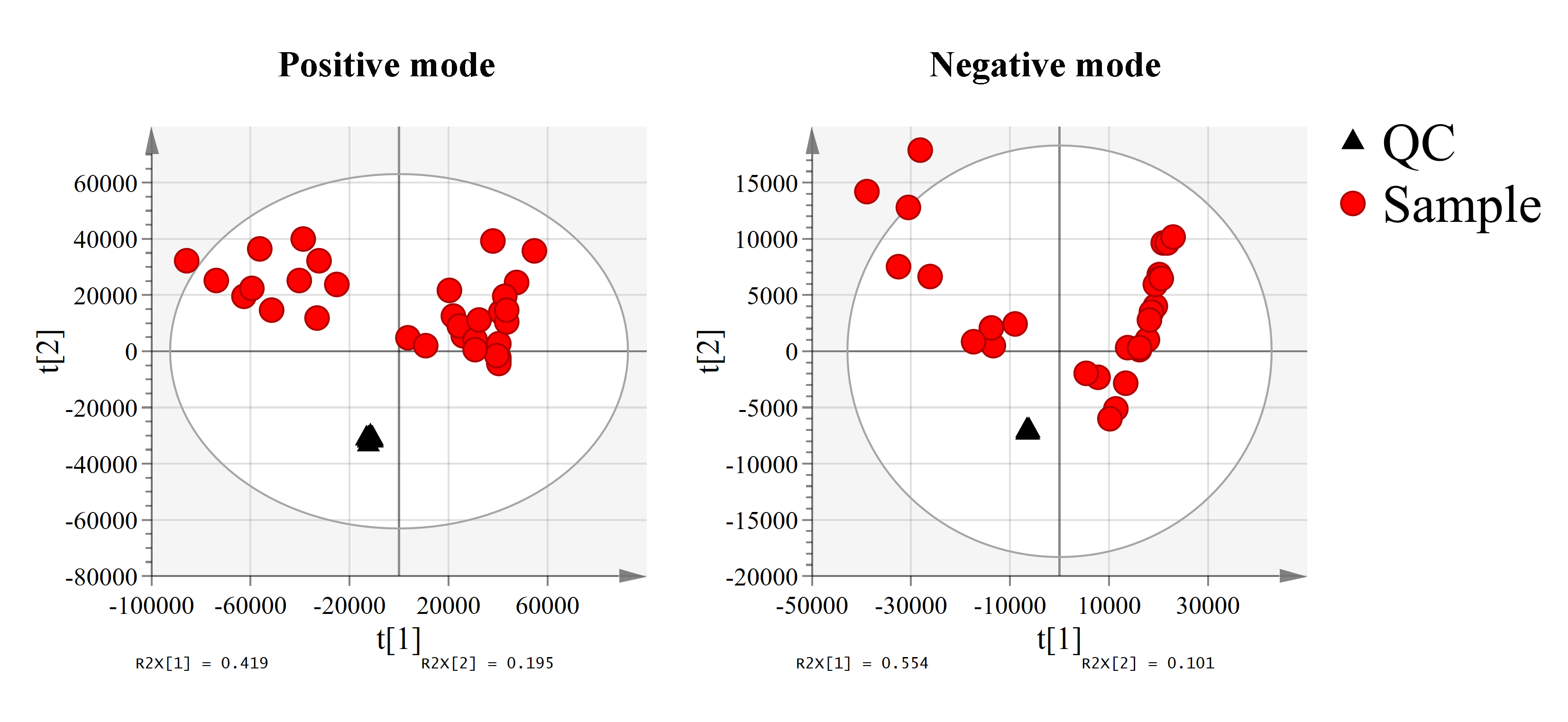
**Fig. S14** PCA of quality control samples and experimental samples based on ion intensity.


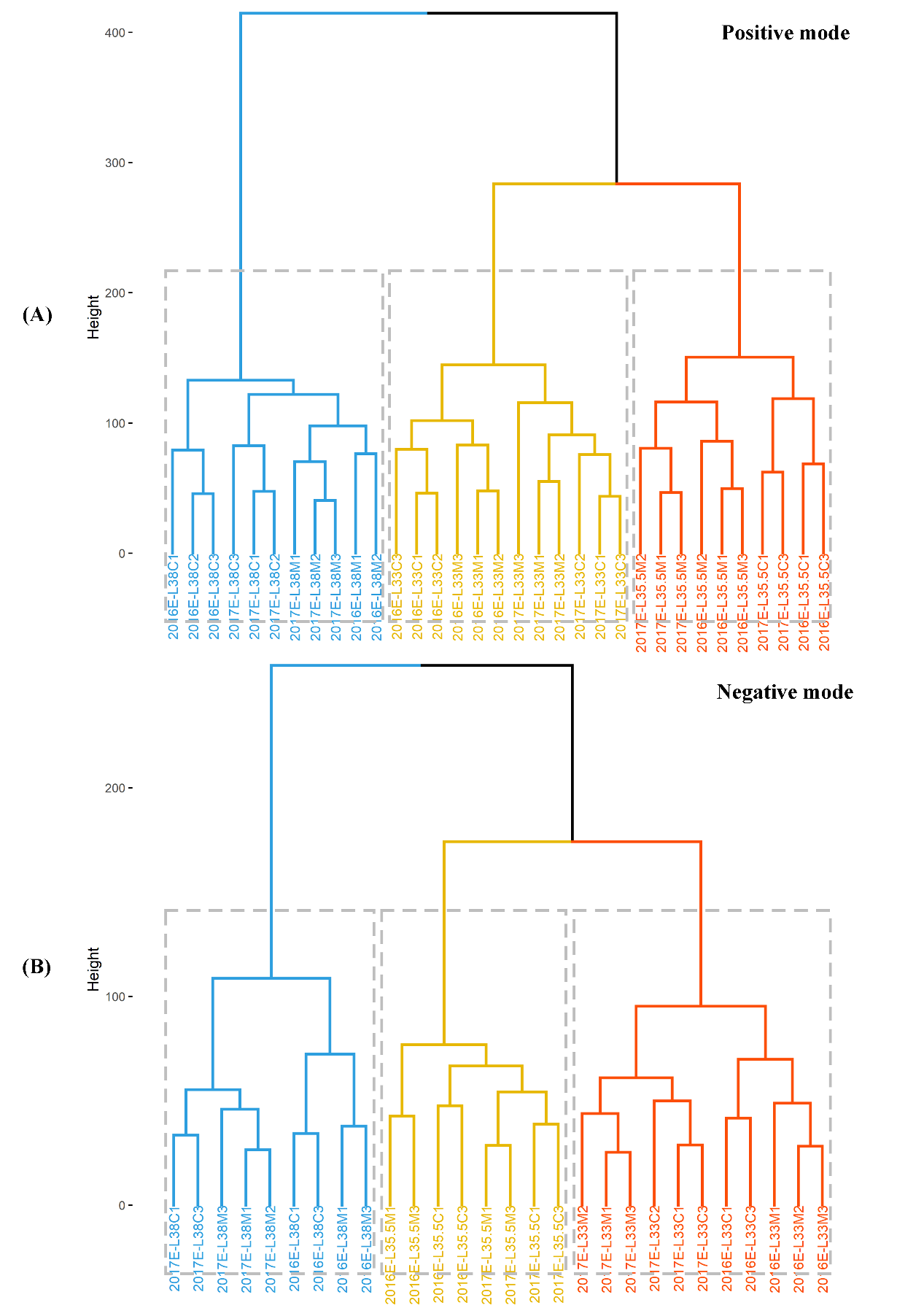
**Fig. S15** Clustering analysis based on ion intensity under positive mode (A) and negative mode (B).
